# Innovative application of magnetically modified bovine horn as a natural keratin resource in the role of value-added organocatalyst†

**DOI:** 10.1039/d1ra09327d

**Published:** 2022-06-06

**Authors:** Atefeh Darvishi, Hadi Bakhshi, Akbar Heydari

**Affiliations:** Chemistry Department, Tarbiat Modares University Tehran 14155-4838 Iran; Department of Life Science and Bioprocesses, Fraunhofer Institute for Applied Polymer Research IAP Geiselbergstraße 69 14476 Potsdam Germany heydar_a@modares.ac.ir

## Abstract

This study presents the conversion of bovine horn powder (BHP) as an available and low-cost waste material to a value-added highly recyclable catalyst. This green catalyst was prepared through the immobilization of BHP, as a natural keratin resource, on the magnetic Fe_3_O_4_ nanoparticles. The successful preparation of the catalyst was fully investigated using Fourier transform infrared, X-ray diffraction, and energy-dispersive X-ray spectroscopies as well as field emission scanning electron microscopy, vibrating sample magnetometry, and thermogravimetry. The catalytic efficiency of the prepared magnetic organocatalyst was evaluated in the synthesis of a large series of amide derivatives through the solvent-free transamidation reaction of different amides and amines with yields of 75–96%.

## Abstract 2

The conversion of bovine horn powder as an available and low-cost waste material to a value-added recyclable organocatalyst for transamidation reaction.

## Graphical abstract

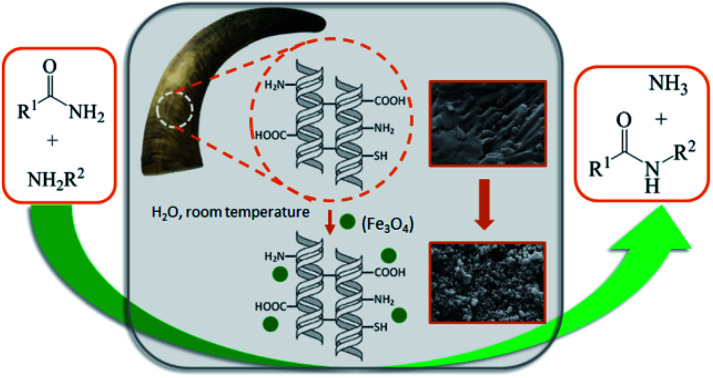

## Introduction

The amide linkage as an important functional group has received considerable interest because of its useful applications in numerous chemicals and natural products. A large number of biological and synthetic polymers, *e.g.* proteins, peptides, alkaloids, nylon, as well as top-selling drugs, *e.g.* valsartan, diltiazem, and captopril, possess necessarily at least one amide functionality.^1^ Hence, any attempt to reach the effective and practical preparation of amide groups could be an admirable achievement in synthetic organic chemistry. The traditional and simplest method for the synthesis of amide bonds involved direct condensation of carboxylic acids and amines. However, such an amidation process was performed in extremely harsh reaction conditions (high temperature) to avoid the unreactive carboxylate ammonium salt formation.^2,3^ Thus, various methods have been developed to create the pre-activation of carboxylic acid which owns far more active derivatives. All these ways have revealed the benefit of mild reaction conditions on account of replacing better leaving-groups in carboxylic acid moiety instead of OH group.^4,5^ These approaches have been effectively employed for some chemical synthesis, *i.e.* peptides, but some limitations such as low atom economy, low reactivity, and tedious work-up procedures have still remained.^6^ Transamidation reaction as one of the most straightforward and valuable methods has acquired a great deal of attention for the formation of amide bonds. Despite using inexpensive and abundant starting materials such as amides and amines, no one has not turned a blind eye to some difficulties such as long reaction time besides high temperature. Therefore, the application of different catalyzed methods using metals, transition metals, biocatalyst, and organocatalyst in transamidation has been studied by many researchers over the last years.^7–11^ To date, numerous catalysts have been used to facilitate the transamination process. Compared with different mentioned catalysts, organocatalysts have been considered by chemists. These are usually stable, waste-free, environment-friendly, easily handled, less expensive, and highly efficient materials, and can be applied using less demanding reaction conditions, like rigorously anhydrous or anaerobic conditions. Amino acids, peptides, vitamins, ureas, carbenes, and phase-transfer agents are some effective organic molecules that are utilized as an organocatalyst in many chemical syntheses.^8,12^ In organocatalysed reactions, the separation and reuse of catalysts are problematic due to the trouble in the recovery of catalysts from the reaction mixture. As a result, by immobilization of the organocatalysts on a solid inert support, such challenges could be solved with ease.^13^

Magnetic nanoparticles, as one of the most ideal and practical solid supports, have been widely applied for many reactions. Magnetite not only has a vastly high active surface to immobilize different ligands and metals but also is separated from the reactions mixtures by an external magnet instead of centrifugation or filtration as relatively difficult manners.^8–10,14–16^ Being well aware of such benefits, many researchers have focused on this area recently. For instance, Varma *et al.*^16^ synthesized a magnetic organocatalyst based on glutathione *via* a simple sonication instrument at room temperature and deployed it successfully for the Paal–Knorr reactions, aza-Michael reactions, and pyrazole synthesis. Kazemi Miraki *et al.*^8^ developed a supported guanidine acetic acid on the magnetic nanoparticles as an organocatalyst for transamidation of different carboxamides with amines under no solvent conditions. Moreover, Gawande *et al.*^15^ described utilizing l-cysteine-based magnetic organocatalyst as a heterogeneous organocatalyst to prepare amino carbonyl compounds. This reaction reflected the yields in the good range under ambient conditions. In the mentioned organocatalysts plus other amino acid-based ones, the most common proposed mechanism pathway in transamidation reaction is the activation of the amide group through the H-bond formation.^17^ Therefore, finding compounds possessing such behaviour in the role of catalyst could be a praiseworthy attempt, especially by considering the environmental aspects.

Bovine horn is a natural by-product of meat and food industries that is discarded as waste material in large quantities. This consists of two distinct parts; a bony structure as the inner core and a keratinous sheath as the outer layer.^18^ Keratin is a durable fibrous protein, with high resistance and low density as well as rich in cysteine amino acid compared with other common ones (7–20% of the total amino acid residues).^18,19^ Keratin is originally divided into two main classes, soft and hard, according to the content of sulfur content. In hard keratin such as nails, hair, horn, or hoof, a number of disulfide bonds as crosslinking bridges lead to high toughness, stiffness and strength compared with soft keratin like skin and callus.^18,19^ The horn keratin is a hard α-keratin with a crystalline fibre phase containing microfibrils with α-helical structure and an amorphous matrix phase madding up of microfibrils with non-helical structure and other morphological components.^18,19^ The keratin fibres are stacked together parallel to the growth direction and form a lamellar structure.^18,19^ During the past decades, using keratin-based natural materials has been widely considered because of the diverse amino acids present. However, most investigations belong to the keratin absorption capability for metals and other pollutants besides biomedical applications.^20–22^ Resultantly, we decided to emphasize the potency of waste materials in the role of value-added products.^14,39–41^ Bovine horn powder (BHP) as a low-cost and abundant material could be a suitable alternative for synthetic and high-priced organocatalyst.

In the current report, for the first time, we have utilized a beneficial and environmentally friendly magnetic organocatalyst based on BHP as a biomass waste disposal and resource recovery. The catalytic activity of this low-cost and simple-prepared magnetic organocatalyst has been evaluated by the synthesis of amide derivatives *via* transamidation reaction. In comparison with other catalysts in the transamidation process, this new green organocatalyst has revealed outstanding and promising results.

## Results and discussion

### Catalyst preparation

The present study aimed to utilize low-cost and abundant BHP as natural keratin-rich waste material with potential organocatalysis application as well as to immobilize magnetic nanoparticles for the facilitate separation of the final catalyst from the reaction media. The keratin-rich BHP possesses many amino acids^23^ with functional groups such as –COOH, –OH, –NH_2_, –SH, *etc.*, that can be modified with magnetite nanoparticles and then use as sufficient organocatalyst through the H-bonding formation.

Many purification methods for extracting keratin from BHP have been presented such as hydrolysis, reduction, oxidation or extracting by ionic liquids.^19^ The soluble keratin doesn't have a three-dimensional structure, which results in poor mechanical stability and processability. Therefore, here, BHP was prepared by collecting the relatively soft and thin layer of the horns' surface and grinding it into smaller pieces, without any further purification.

**Scheme 1:**
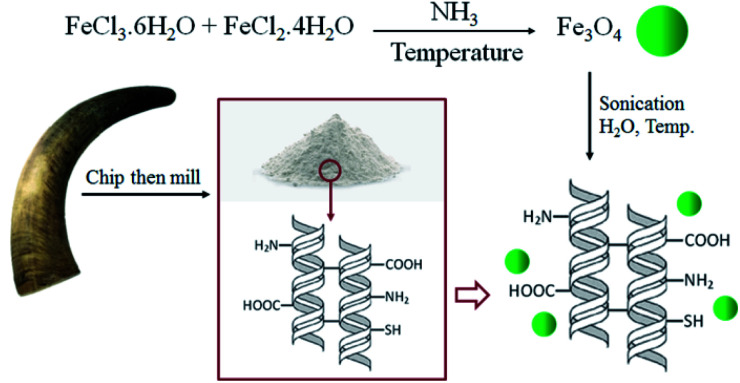
Preparation of BHP@Fe_3_O_4_ organocatalyst.

The simple method for the immobilization of the magnetic Fe_3_O_4_ nanoparticles into BHP (BHP@Fe_3_O_4_) is illustrated in Scheme 1. The magnetite Fe_3_O_4_ nanoparticles were prepared by the co-precipitation method using iron(ii) and iron(iii) chloride salts in aqua media and ammonia to control pH during the process. Afterwards, these nanoparticles were immobilized on the surface of BHP to prepare a stable magnetic heterogeneous organocatalyst with a superparamagnetic activity.

### Catalyst characterization

**Fig. 1:**
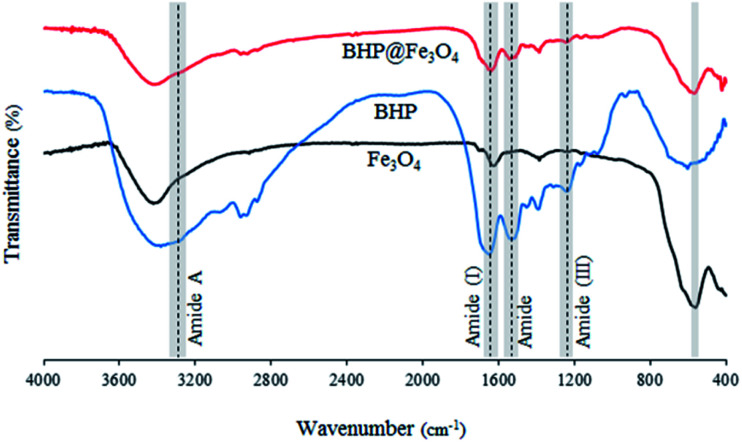
FTIR spectra of BHP, Fe_3_O_4_ nanoparticles, and BHP@Fe_3_O_4_ organocatalyst.

The Fourier transform infrared (FTIR) spectra of BHP, Fe_3_O_4_ nanoparticles, and BHP@Fe_3_O_4_ organocatalyst are illustrated in Fig. 1. The FTIR spectrum of BHP indicated the peaks related to the stretching vibration of hydrogen-bonded N–H bonds (amide A) around 3300–3400 cm^−1^, as well as, the stretching vibration of the C=O group at 1633 cm^−1^, the bending vibration of N–H bond at 1510 cm^−1^, and the stretching vibration of C–N bond at 1220 cm^−1^ belong to amide I, amide II and amide III, respectively. All four maintained peaks have been known as characteristic bonds of keratin structure.^24^ In the FTIR spectrum of BHP@Fe_3_O_4_, all amide typical bonds (amide A, amide I, amide II, and amide III) were repeated with no change in shape and position. This demonstrated that the peptide bonds in magnetic organocatalyst were not strongly influenced during the magnetization process. Additionally, the absence of peaks at 1317 cm^−1^, 1170 cm^−1^, and 1124 cm^−1^ in the FTIR spectra of BHP and BHP@Fe_3_O_4_, relating the breakage of disulfide bonds and the formation of sulfide bonds as well as sulfur-containing derivatives, demonstrated the stability of the keratin backbone during the chipping and milling processes as well as the modification with magnetic nanoparticles.^25^ According to the spectrum of Fe_3_O_4_ nanoparticles, the stretching vibration of Fe–O appeared at 556 cm^−1^. The existence of this bond in the BHP@Fe_3_O_4_ spectrum with a minimum decrease in intensity proved that the Fe_3_O_4_ nanoparticles were successfully grafted to O and N atoms of amino acids in BHP.

**Fig. 2:**
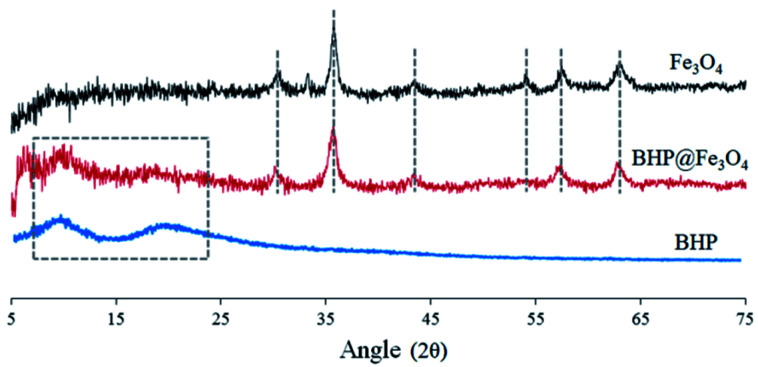
XRD spectra of BHP, Fe_3_O_4_ nanoparticles, and BHP@Fe_3_O_4_ organocatalyst.

The X-ray diffraction (XRD) spectra of BHP, Fe_3_O_4_ nanoparticles, and BHP@Fe_3_O_4_ organocatalyst to recognize their crystal phases are illustrated in Fig. 2. In the XRD pattern of BHP, the large diffraction angles (2*θ*) at around 9 and 20 are attributed to the α-helix and the β-sheet structures, respectively.^26^ The mentioned diffraction peaks were also observed in the BHP@Fe_3_O_4_ XRD pattern. Regarding magnetite nanoparticles, all shown 2*θ* at 30.18, 35.51, 42.80, 53.97, 57.03, and 62.73 which are corresponding to (220), (311), (400), (422), (511), and (440) are six typical peaks of a standard Fe_3_O_4_ crystal with a spinel structure. Comparing the mentioned peaks in bare Fe_3_O_4_ nanoparticles with the BHP@Fe_3_O_4_ organocatalyst indicated the presence of all peaks with no change in the position. It could mean that the surface modification process did not have any significant impact on the crystalline structure of Fe_3_O_4_ nanoparticles and BHP in the final organocatalyst which possesses properties of its two components at the same time.

**Fig. 3:**
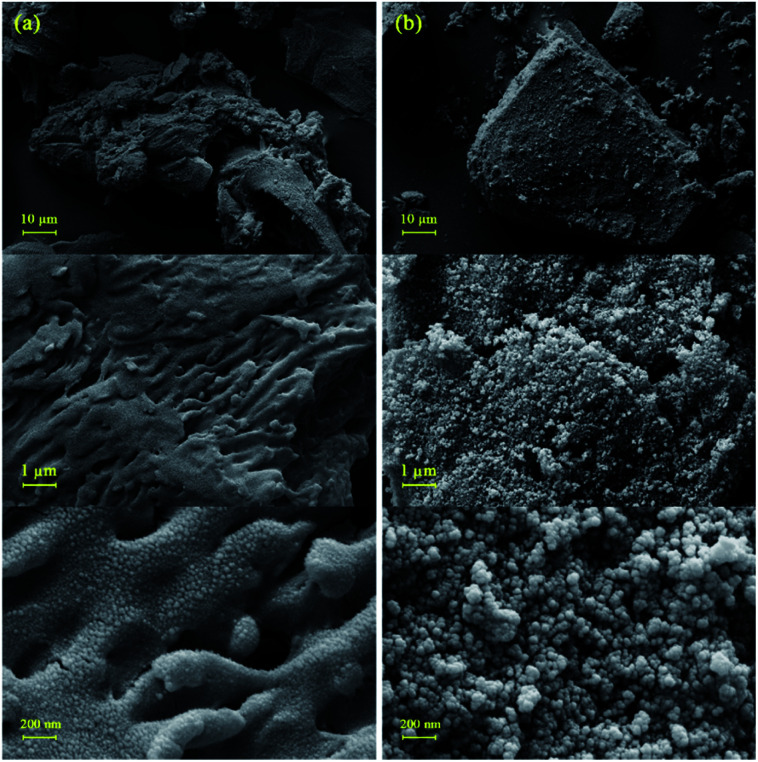
FE-SEM image of BHP (a) and BHP@Fe_3_O_4_ organocatalyst (b).

The surface morphology and particle size of the samples were fully characterized by field emission scanning electron microscopy (FE-SEM). Fig. 3a displays the surface morphology of BHP after mechanical chipping and subsequently milling to reach minimum particle size. A formless and irregular shape of individual particles with some laminate structures was distinctly revealed. The exhibition of a layered structure with a rippled shape involving several flattened dead keratin-filled keratinocytes was expected according to the previous reports.^27,28^ The particle size was approximately between 50–100 μm. In more detailed images of BHP, the flattened keratinized cell with a relatively rough and uneven surface was exhibited as labyrinth-like wavy morphology. It seems that such nanostructure is intermediate filaments embedded in the matrix.^27,29^

After the immobilization of the BHP with Fe_3_O_4_ nanoparticles, the main structure of BHP remained constant while uniform spherical magnetic nanoparticles were added to the surface of the keratinous substrate. These nanoparticles appeared size range of ∼30 nm (Fig. 3b).

**Fig. 4:**
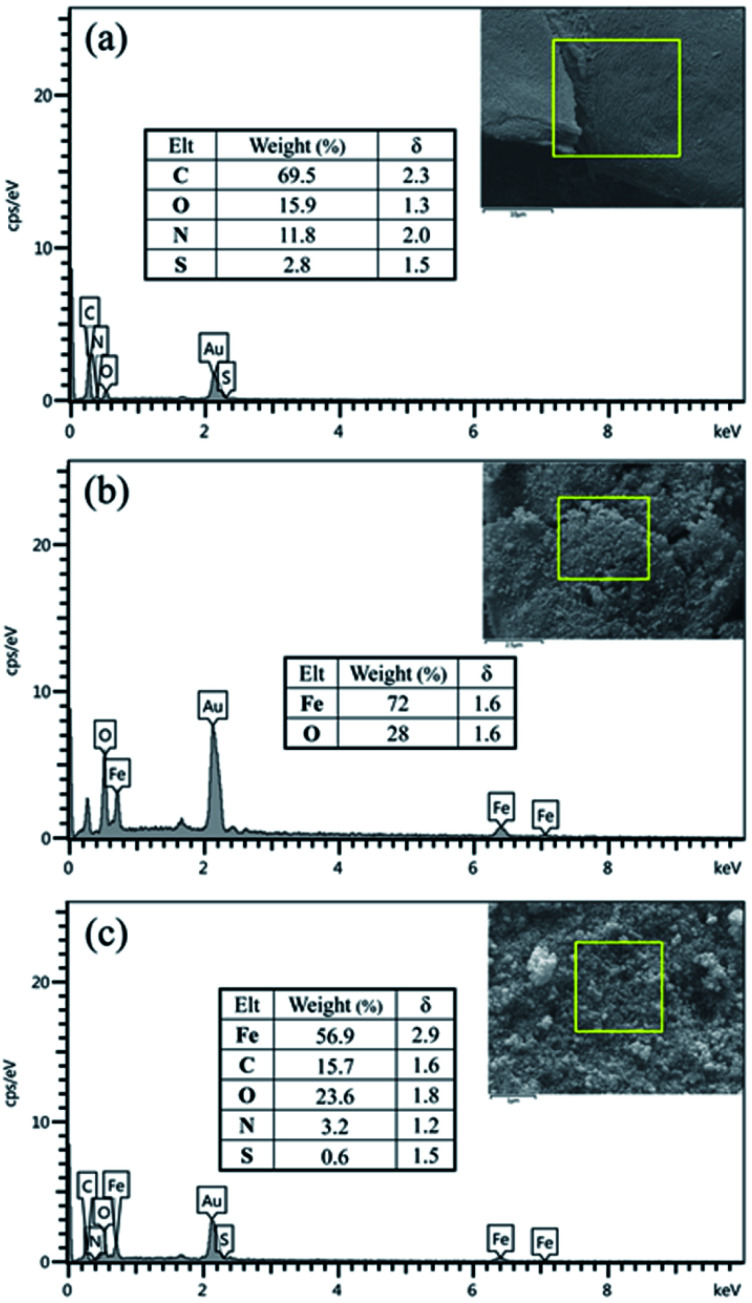
EDX spectra of BHP (a), (b) Fe_3_O_4_ nanoparticles (c) and BHP@Fe_3_O_4_ organocatalyst.

The elemental composition of BHP, Fe_3_O_4_ nanoparticles, and BHP@Fe_3_O_4_ organocatalyst was evaluated by energy-dispersive X-ray (EDX) analysis (Fig. 4). As expected, all EDX patterns represented the corresponding elements of their chemical compositions. Moreover, according to the BHP@Fe_3_O_4_ EDX pattern, the organocatalyst was composed of C (15.7 w%), O (23.6 w%), N (3.2 w%), S (0.6 w%), and Fe (56.9 w%) elements. It could mean that the observed reasonable ratio in individual elements content of BHP and Fe_3_O_4_ nanoparticles in the final catalyst remained constant. The magnetization process of BHP had no remarkable emphasis on its amino acid-rich structure and did not occur decomposition on the keratin horn backbone.

**Fig. 5:**
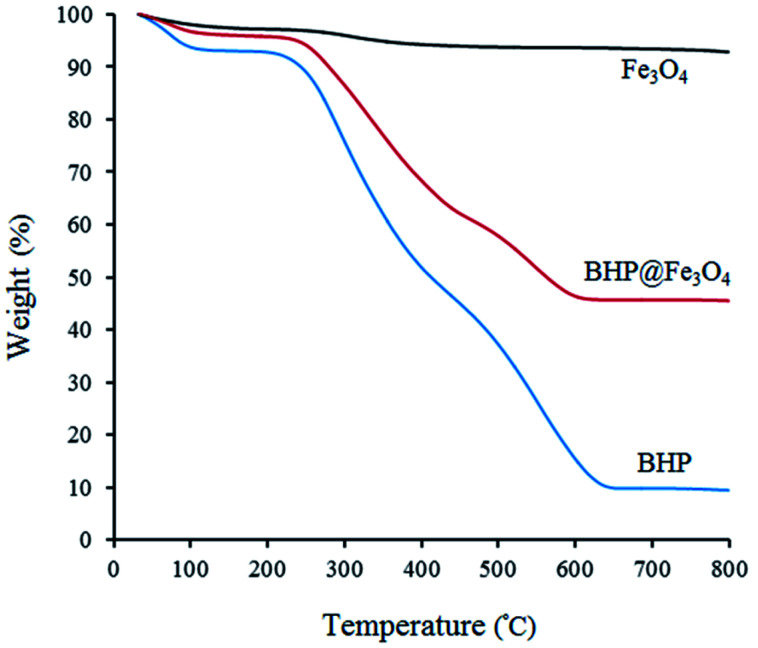
TGA curves of BHP, Fe_3_O_4,_ and BHP@Fe_3_O_4_ organocatalyst.

The thermal stability of BHP, Fe_3_O_4_ nanoparticles, and BHP@Fe_3_O_4_ organocatalyst was investigated *via* thermogravimetric analysis (TGA) by heating samples from room temperature to 800 °C with a rate of 10 °C min^−1^ (Fig. 5). As it is shown in all curves, the first weight loss below 100 °C is attributed to the evaporation of the absorbed water molecules.^14,30^ The second mass loss at the temperature range of 200–450 °C in BHP and BHP@Fe_3_O_4_ catalyst is due to the degradation of organic residues and keratin matrix denaturation. The third mass loss occurred at the temperature range of 450–650 °C for BHP and 450–600 °C for BHP@Fe_3_O_4_ organocatalyst. This mass loss can be attributed to the decomposition of keratin and the subsequent release of volatile compounds like H_2_S, CO, CH_4,_ and HCN. The carbonized residue was achieved after heating up to 700 °C.^30,31^ The immobilization of magnetic nanoparticles to the surface of BHP led to an increase in the residual char for the BHP@Fe_3_O_4_ catalyst in comparison with BHP. Finally, a proper magnetic biocatalyst was successfully synthesized with sufficient resistance to the high temperatures (120 °C), which are applied during the current transamidation reaction.

**Fig. 6:**
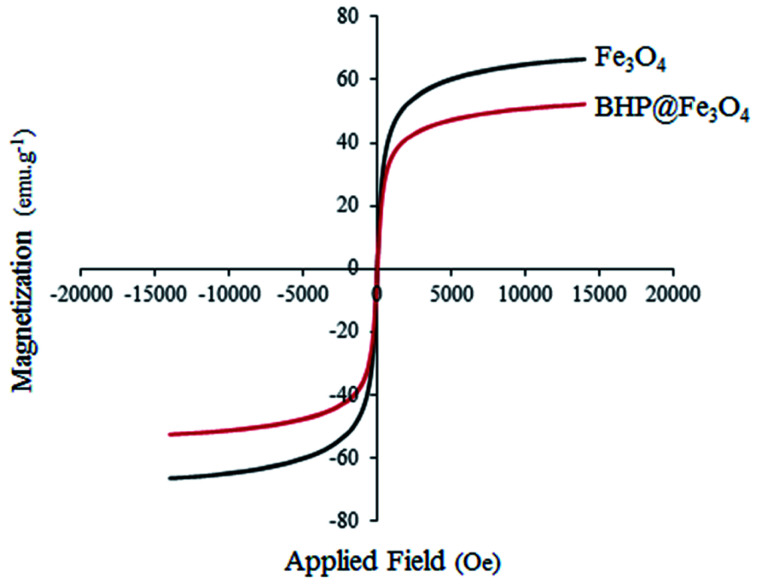
VSM spectrum of Fe_3_O_4_ nanoparticles and BHP@Fe_3_O_4_ organocatalyst.

The magnetic properties of Fe_3_O_4_ nanoparticles and BHP@Fe_3_O_4_ catalyst were thoroughly characterized by vibrating sample magnetometry (VSM) at room temperature with the field sweeping from −2000 to 2000 Oe (Fig. 6). The saturated magnetization (*M*_s_) values for Fe_3_O_4_ nanoparticles and BHP@Fe_3_O_4_ organocatalyst were 66.5 and 52 emu g^−1^, respectively. The *M*_s_ value of organocatalyst is lower than that of magnetic nanoparticles due to the modification of Fe_3_O_4_ with BHP as non-magnetic particles. This could demonstrate the successfully formed bond between two components in the final catalyst. Meanwhile, the reversible state in the hysteresis loops as well as *M*_s_ values of both curves not only exhibited the superparamagnetic property of the samples but also showed the complete separation of prepared nanocatalyst from reaction system with helping an external magnet with simple.

### Catalytic transamidation reaction

After the comprehensive characterization of the organocatalyst, the activity of the maintained green magnetic catalyst was explored through the transamidation reaction of acetamide with aniline as the model system (Table 1). Therefore, the reaction was optimized regarding the most suitable catalyst amount in the absence of solvent at 120 °C under an Ar atmosphere (Table 1, entries 1–3). The best product yield of 90% was achieved using 20 mg of catalyst, while an increase up to 30 mg does not affect the yield. On the contrary, in less quantity (10 mg) a significant reduction of yield to 70% was obtained (Table 1, entry 3). Meanwhile, the reaction occurred with minimum yield when there is no content of catalyst in the reaction mixture even at harsh temperatures (150 °C, Table 1, entries 4 and 5). To reach the optimum temperature, the reaction mixture was heated under 100, 110, and 130 °C. Although the yields remarkably improved by 15% and 25% units with increasing the temperature from 100 to 110 °C and 120 °C, respectively (Table 1, entries 2, 6, and 7), no such increase occurred at 130 °C (Table 1, entry 8). As control experiments and to understand the real impact of synthesized catalyst, the reaction was performed by the use of BHP and also Fe_3_O_4_ nanoparticles in the role of catalyst (Table 1, entries 9 and 10). Interestingly, in the case of BHP, the yield was similar to that of the magnetic one, while by utilizing magnetite nanoparticles low yield (>30%) was obtained. These results indicated the main catalytic role of functional groups on the surface of horn particles during the transamidation reaction that didn't change under the magnetization process. Finally, to screen the effect of solvents as an important reaction factor, H_2_O, EtOH, CH_3_CN, DMF, DMSO and toluene were applied in reflux conditions. The results demonstrated that the best yield was obtained using toluene (80%) in comparison to other solvents (Table 1, entries 11–16). However, far more outcomes appeared in neat condition (Table 1, entry 2). Thus, using 1 mmol of acetamide and 1.5 mmol of aniline besides the consumption of 20 mg magnetic nanocatalyst at 120 °C in the absence of solvent is the optimal condition for the current transamidation reaction.

**Table 1:** Optimization of transamidation reaction conditions^a^

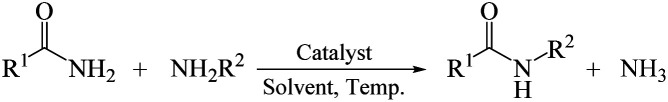				
Entry	Catalyst (mg)	Solvent	Temperature (°C)	Yield^b^ (%)
1	30	—	120	90
**2**	**20**	**—**	**120**	**90**
3	10	—	120	85
4	—	—	120	<5
5	—	—	150	<10
6	20	—	100	65
7	20	—	110	80
8	20	—	130	90
9	20^c^	—	120	90
10	20^d^	—	120	<30
11	20	H_2_O	Reflux	30
12	20	EtOH	Reflux	45
13	20	CH_3_CN	Reflux	70
14	20	DMF	Reflux	35
15	20	DMSO	Reflux	50
16	20	Toluene	Reflux	80

a Reaction condition: acetamide (1 mmol), aniline (1.5 mmol), solvent (2 mL), under Ar atmosphere.

b Isolated yield.

c BHP.

d Fe_3_O_4_ nanoparticles.

After achieving the optimum conditions, the generality of the transamidation reaction was studied with different amides and amines. Therefore, a series of amide derivatives were synthesized (Table 2). The transamidation of acetamide with a variety of aniline derivatives as well as benzylamine was performed with good to excellent yields (76–95%). In the case of aniline, the presence of an electron-withdrawing group on the benzene ring (Table 2, entry 4) led to far higher yields in comparison to electron-donating groups (Table 2, entries 1–3). However, the use of benzylamine reflected a better yield (Table 2, entry 5). Subsequently, benzamide derivatives were reacted with different primary and secondary amines to obtain corresponding amides. As it is obvious, the best yield of 96% was obtained by applying benzylamine (Table 2, entry 6). Opposite of the aniline case, the existence of electron-withdrawing groups on the benzylamine ring, reduced the yields up to 78% (Table 2, entry 7). Entering electron-withdrawing groups on benzamide moiety resulted in a decrease in yields (Table 2, entries 10–12). In the case of aliphatic amines, a higher yield of 91% was obtained *via* primary propylamine (Table 2, entry 13) compared with secondary amines (Table 2, entries 14–15). To further extend the scope of this methodology besides investigating the role of different groups on the benzamide ring, the catalytic transamidation reaction was performed *via* 1-phenylethylamine and various benzamides. As a result, benzamides that bearded electron-donating groups like methoxy and methyl (Table 2, entries 17–18) revealed more suitable results compared with electron-withdrawing groups (Table 2, entry 19). Regarding benzamide derivatives with aniline, similar to benzylamine, the presence of the electron-donating group achieved better yields compared to electron-withdrawing groups (Table 2, entries 20–24). Finally, our study was focused on the transamidation of urea and thiourea with aniline and 1-phenylethylamine while corresponding target products revealed good yields (Table 2, entries 25–27).

**Table 2:** Transamidation reaction of primary amides with amines using BHP@Fe_3_O_4_ organocatalyst^a^

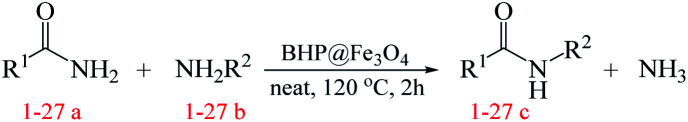				
Entry	Amide	Amine	Product	Yield (%)
1	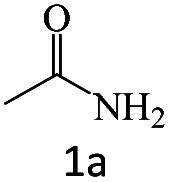	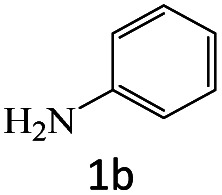	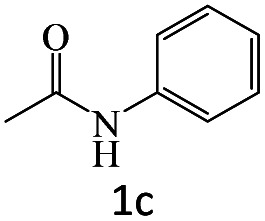	90
2	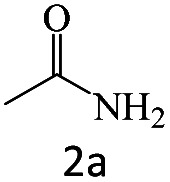	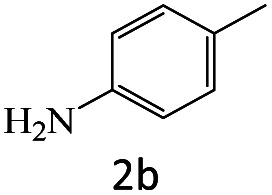	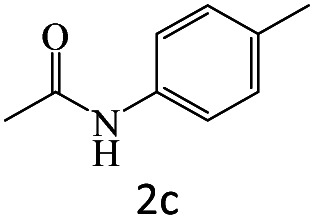	84
3	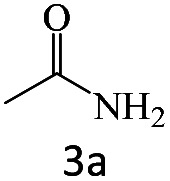	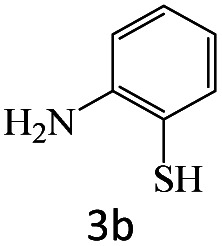	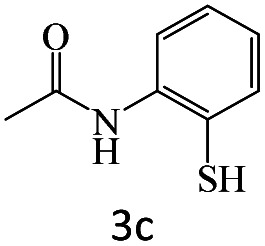	76
4	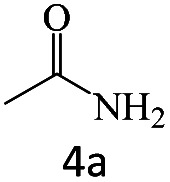	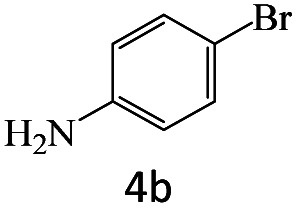	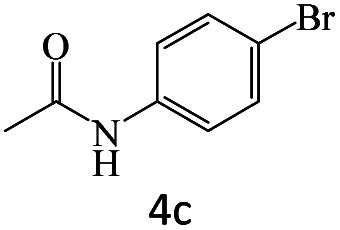	92
5	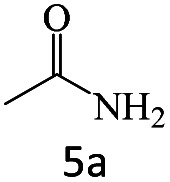	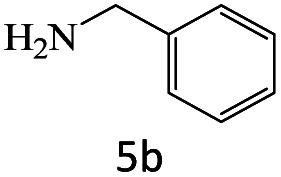	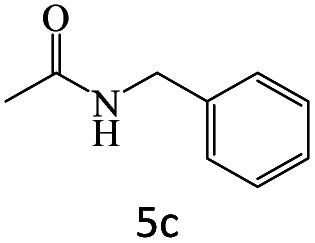	95
6	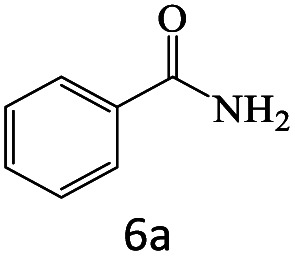	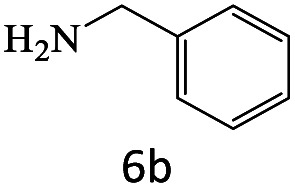	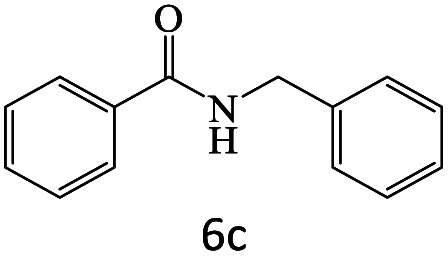	96
7	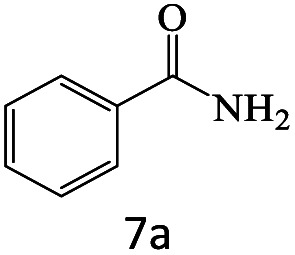	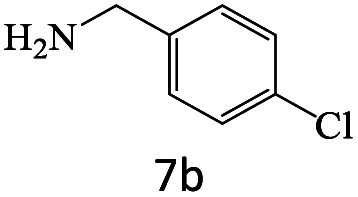	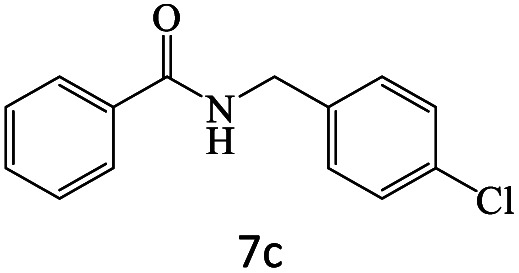	78
8	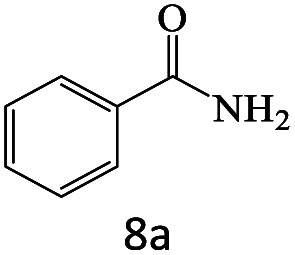	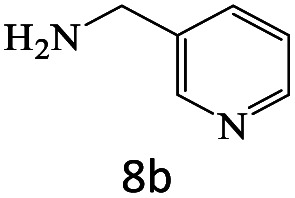	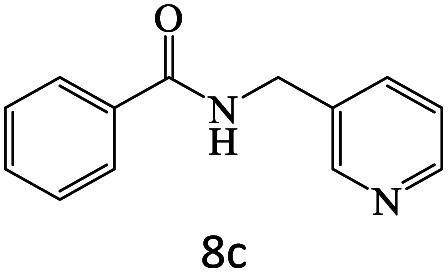	75
9	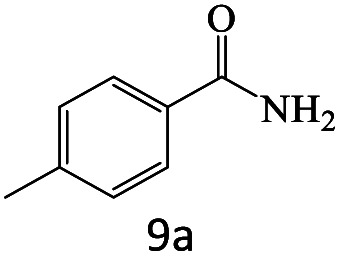	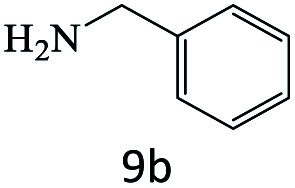	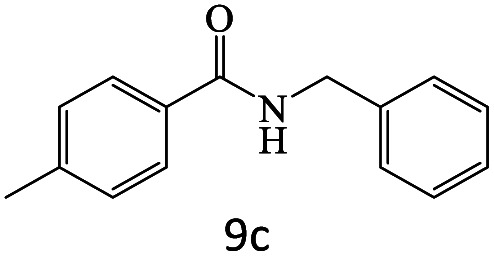	90
10	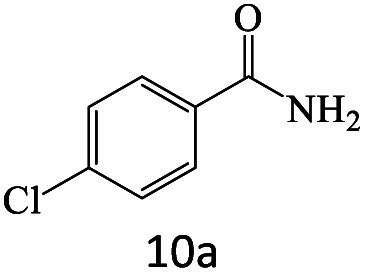	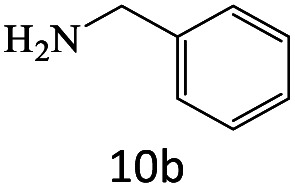	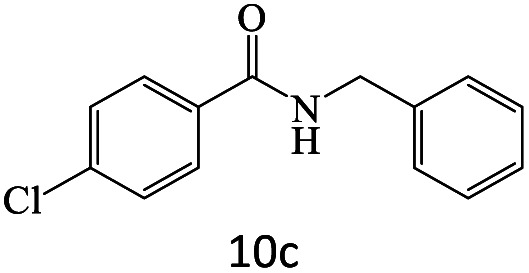	87
11	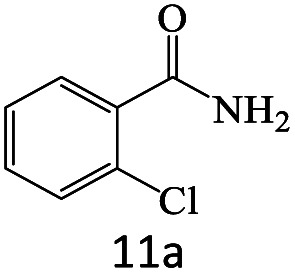	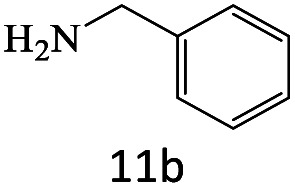	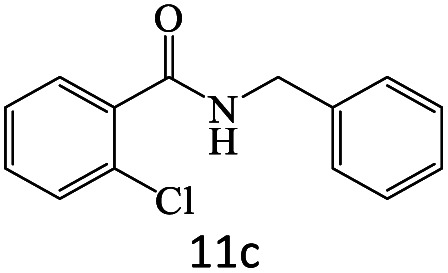	79
12	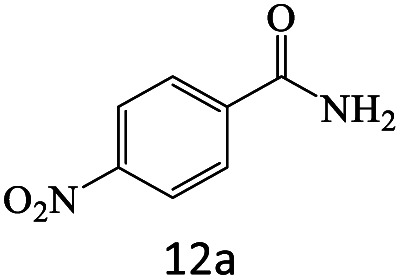	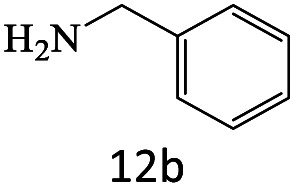	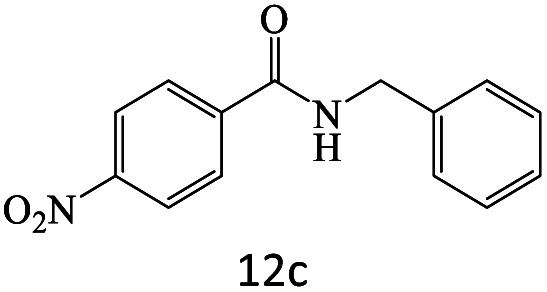	79
13	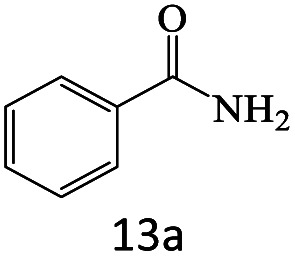	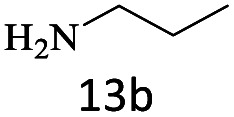	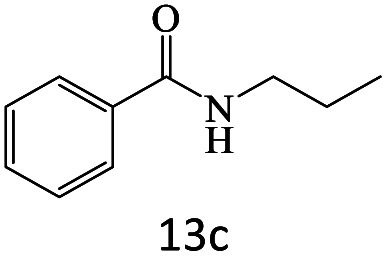	91
14	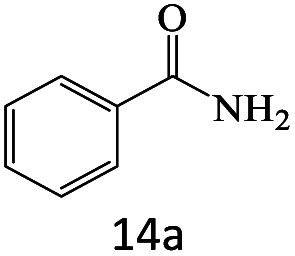	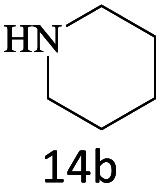	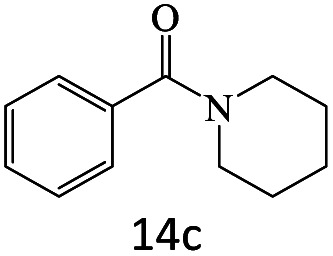	70
15	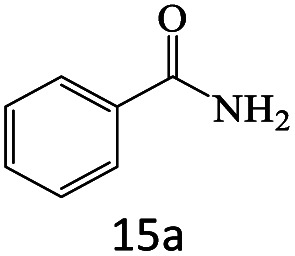	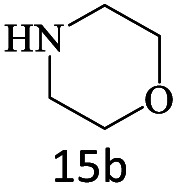	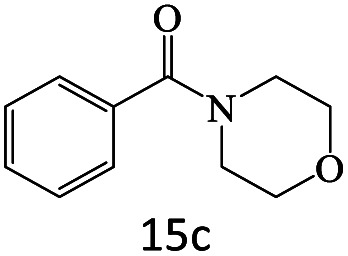	80
16	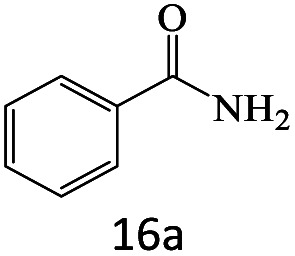	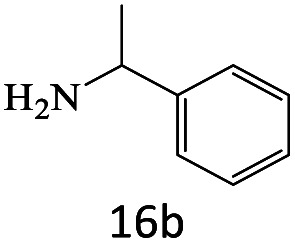	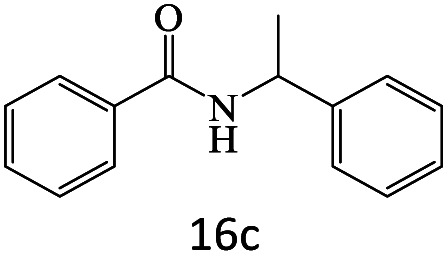	80
17	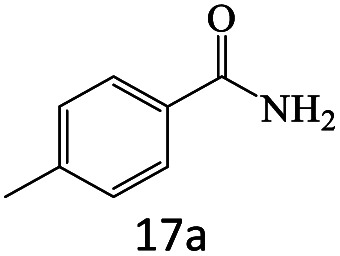	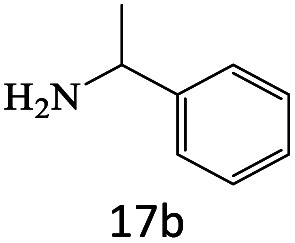	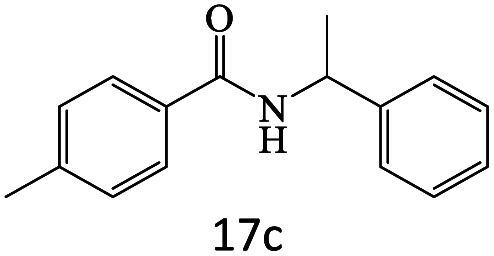	83
18	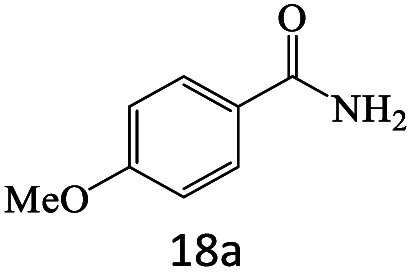	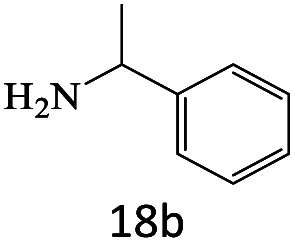	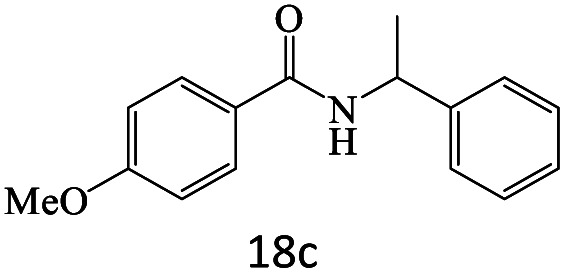	87
19	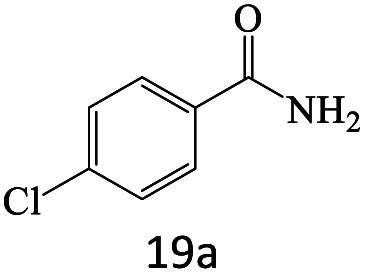	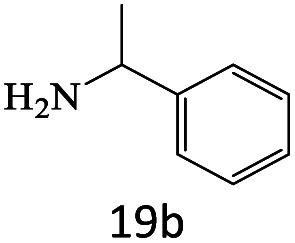	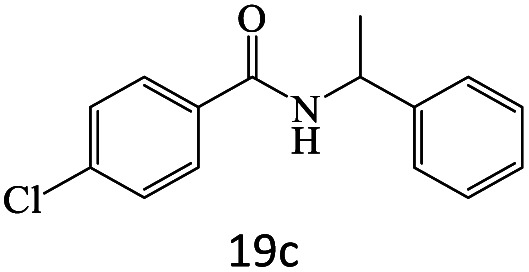	78
20	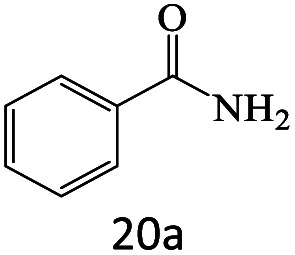	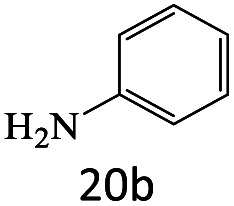	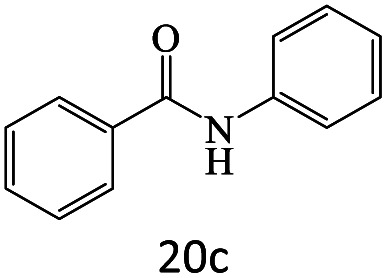	81
21	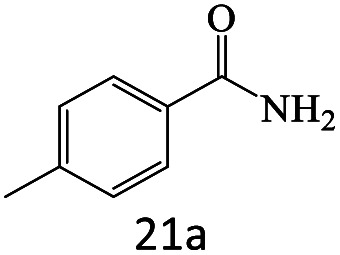	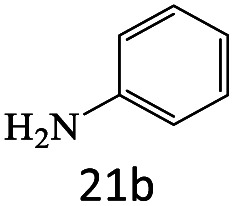	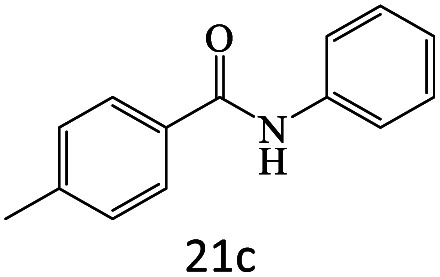	76
22	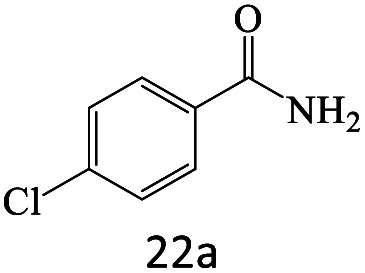	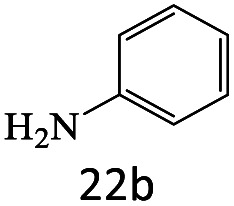	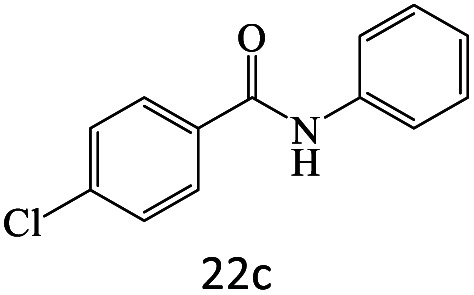	75
23	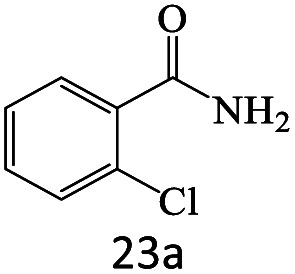	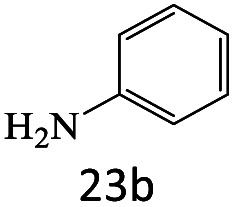	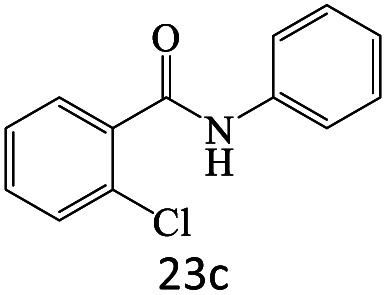	71
24	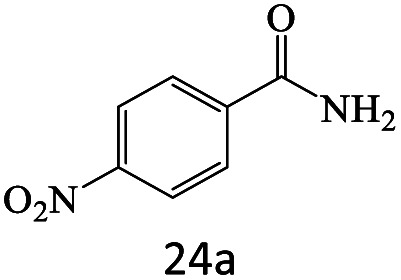	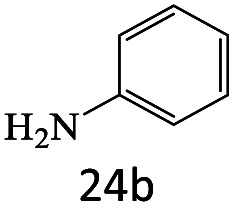	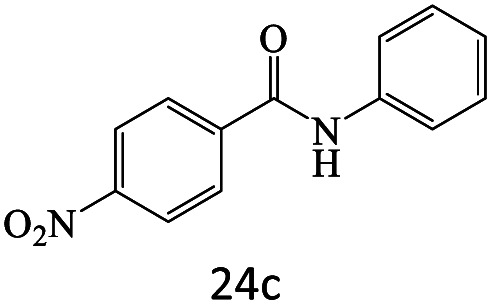	72
25	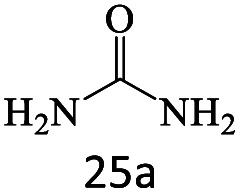	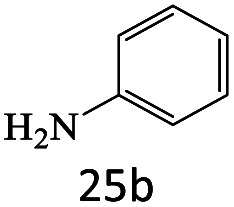	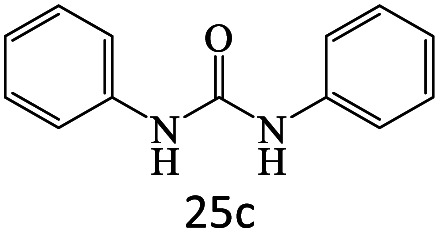	86
26	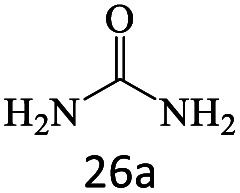	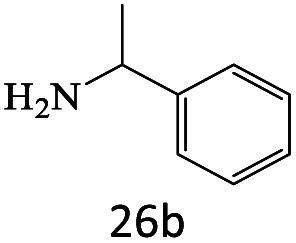	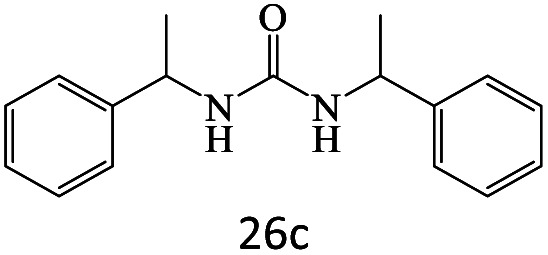	90
27	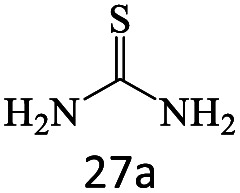	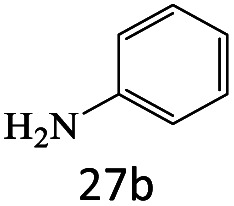	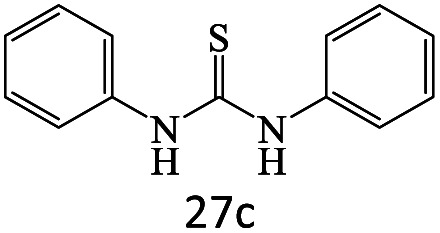	80

a Reaction condition: acetamide (1 mmol), aniline (1.5 mmol), BHP@Fe_3_O_4_ (20 mg), 120 °C under Ar atmosphere.

**Scheme 2:**
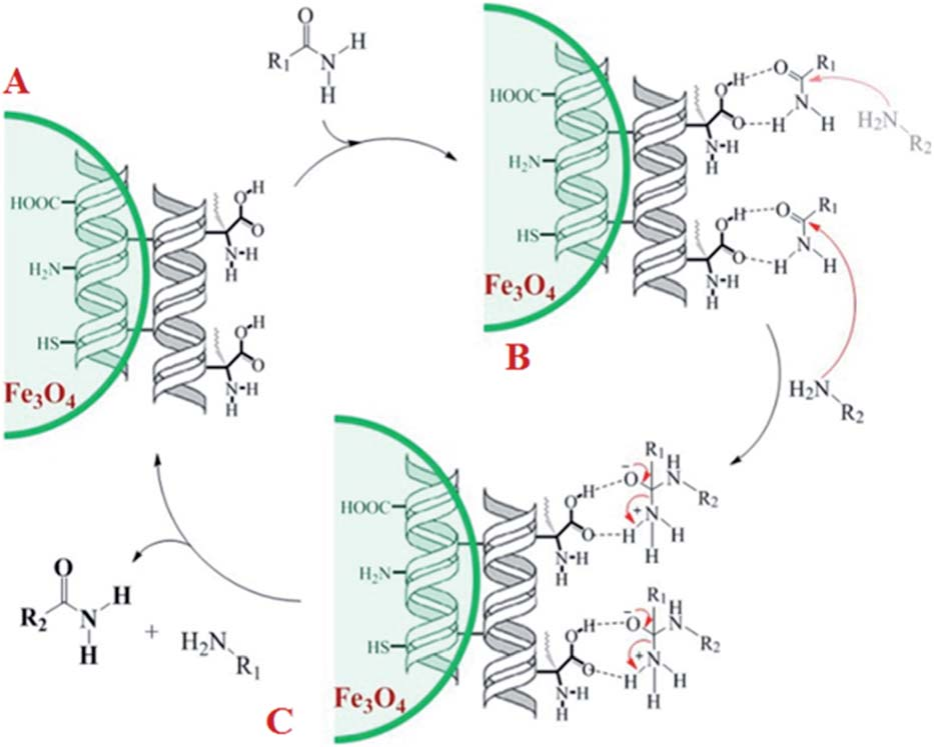
Proposed mechanism for transamidation reaction.

A plausible mechanism for transamidation reaction catalyzed by magnetic organocatalyst was proposed in Scheme 2. Based on the previous reports, the keratin structure of the bovine horn is composed of a large number of amino acids such as glutamine, glycine, serine, asparagine, arginine, and cysteine with different functional groups.^23^ Therefore, despite the immobilization of these particles by magnetic nanoparticles, there are adequate numbers of heteroatoms to form hydrogen bonds to facilitate the reaction pathway. As it is shown in Scheme 2, some selective functional groups as typical ones were entered into such catalytic mechanism. In transamidation reaction *via* prepared magnetic organocatalyst, there is a suggested mechanism consisting of three main stages. The first one was the reaction of amide groups with BHP@Fe_3_O_4_ organocatalyst to form the intramolecular hydrogen bonds as the amide activation stage (intermediate B). After that, amine groups directly were attacked to the carbonyl position and then the unstable intermediate was immediately undergone a reversible proton exchange to make more stable another intermediate (C). Eventually, the amide product besides the corresponding amine dissociated from the catalyst backbone, while the organocatalyst regenerated for entering the repeated cycle mechanism.

The efficiency of the BHP@Fe_3_O_4_ organocatalyst in the transamidation reactions was compared to those of other previously reported catalysts (Table 3). The results indicated notable yields in the current work. We attempted to alter the harsh condition like high temperature, long reaction time, and even in some cases the presence of organic solvents to far more moderate ones, while remaining yield at a proper level. Furthermore, considering the waste aspect of BHP in the role of the catalyst, we developed an organocatalyst that can be utilized as a sufficient and cost-effective one in the transamidation reactions.

**Table 3:** Comparison of the efficiency of different catalysts for transamidation reaction^a^

Entry	Catalyst	Reaction condition	Yield (%)	Ref.
1	Sulfated tungstate	Temp.: reflux, 12 h, toluene	88	11
2	Chitosan	Temp.: 150 °C, 36 h, neat	89	32
3	l-Proline	Temp.: 150 °C, 36 h, neat	84	12
4	Diacetoxyiodobenzene	Temp.: 120 °C, 24 h, neat	81	33
5	H-β-Zeolite	Temp.: 130 °C, 24 h, neat	60	34
6	Ionic liquid	Temp.: 120 °C, 21 h, neat	46	35
7	Imidazolium chloride	Temp.: 150 °C, 2 h, DMA	92	36
8	[Ru–NHC] complex	Temp.: 110 °C, 8 h, toluene	94	37
9	Fe-mont	Temp.: reflux, 30 h, toluene	86	38
10	Fe(OH)_3_/Fe_3_O_4_	Temp.: reflux, 10 h, *p*-xylene	70	10
11	Fe_3_O_4_/GAA	Temp.: 120 °C, 8 h, neat	85	14
**12**	**BHP@Fe**_**3**_ **O**_**4**_	**Temp.: 120 °C, 2 h, neat**	**90**	**This work**

a Reagents: amide: acetamide, amine: aniline.

**Fig. 7:**
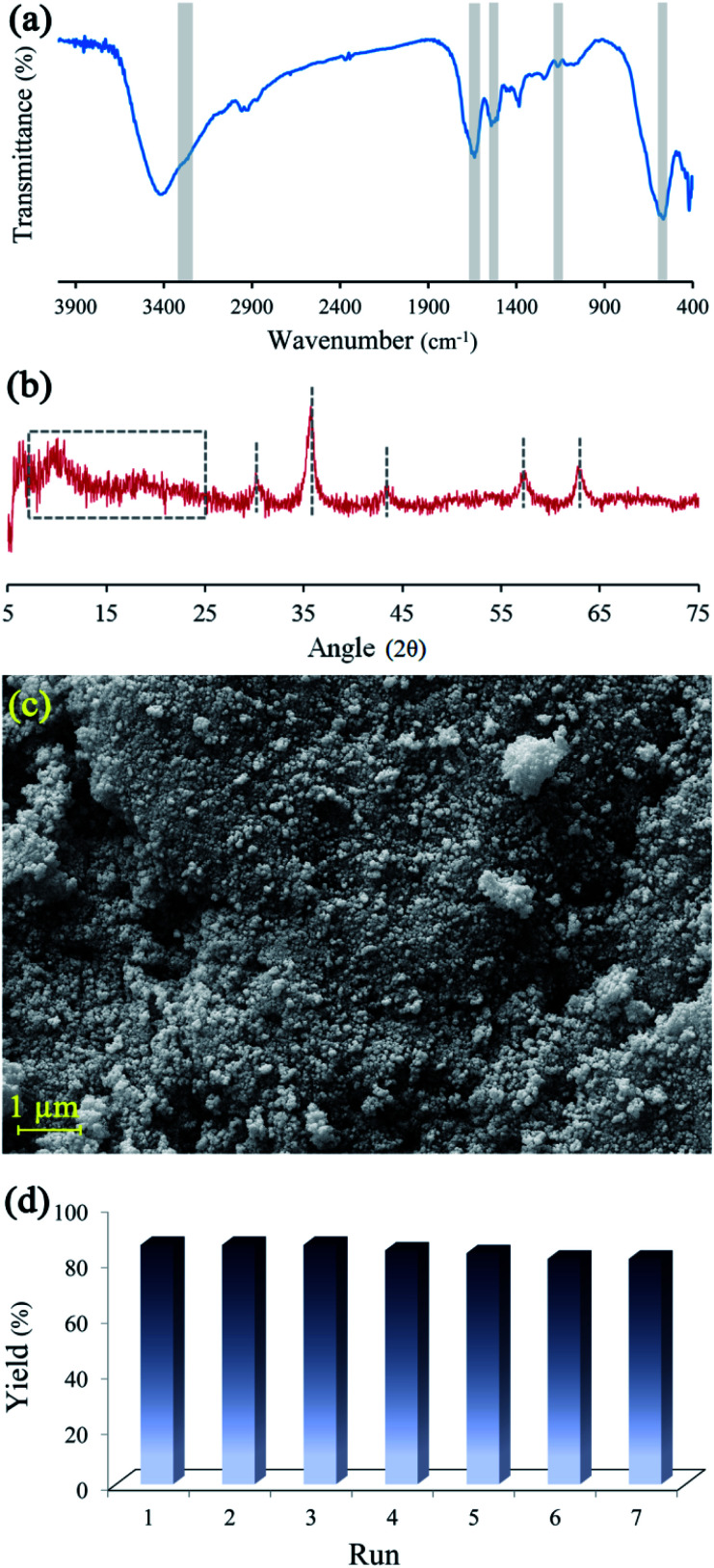
FTIR spectrum (a), XRD spectrum (b), FESEM image (c) of BHP@Fe_3_O_4_ after 7th reaction run and BHP@Fe_3_O_4_ organocatalyst recycling chart (d).

Reusability, as a fundamental property in heterogeneous catalysts, was investigated on BHP@Fe_3_O_4_. The recyclability of magnetic nanocatalyst was studied during the transamidation reaction (Fig. 7). After the reaction, the magnetic catalysts were thoroughly separated from the reaction mixture *via* an external magnet, washed three times with ethanol, and then dried at ambient temperature. Then, the recycled catalyst was characterized by FTIR, XRD, and FESEM techniques to prove the stability of the catalyst during the transamidation and recovering processes. Meanwhile, the recycled catalyst was successfully used seven times without any significant impact on its catalytic activity.

## Experimental

### Materials and instruments

BHP used in this experiment was obtained as waste from a Slaughterhouse in Tehran, Iran. The bovine horns were washed extensively with distilled water to remove impurities. Then, only the relatively soft and thin layer of the horns' surface was grated carefully. Afterwards, the obtained large pieces were cut into smaller ones using a grinder and saved at room temperature for further procedures. Other high purity chemical substances containing reagents and solvents used in the experiment were of analytical grade, were purchased from Sigma-Aldrich and Merck companies, and utilized without further purification.

Infrared spectra were collected by an FTIR spectrometer (Bruker Instruments, model Aquinox 55, Germany) at 400–4000 cm^−1^ wavenumbers using pressed KBr pellets. The surface morphology of the samples was determined using an FE-SEM (Philips XL 30 and S-4160) coupled with an energy dispersive spectroscopy detector (EDS/Oxford Instrumental). The samples were coated with a thin gold layer before microscopy. The crystalline structure of the samples was characterized at room temperature using XRD (Philips X-Pert 1710, Cu K*α*, *α* = 1.78897 Å, voltage: 40 kV, current: 40 mA) in the 2*θ* range of 10° to 90° at a scanning speed of 0.02 s^−1^. TGA was conducted from 25–800 °C under an N_2_ atmosphere and a heating rate of 10 °C min^−1^ using a Netzsch instrument (Germany). The magnetic properties of the sample were obtained by VSM) (MDK Co, Iran). The ^1^H-NMR and ^13^C-NMR spectra were recorded on a Bruker Instrument (model advance 400, Germany) at 250 and 400 MHz in CDCl_3_ using TMS as an internal standard. Thin-layer chromatography (TLC) was carried out on silica gel 254 analytical sheets (Fluka). A Gallenkamp melting point apparatus was utilized to determine the melting points of synthesized derivatives.

### Preparation of Fe_3_O_4_ nanoparticles

Iron oxide nanoparticles were synthesized by chemical co-precipitation of Fe^3+^ and Fe^2+^ ions with a molar ratio of 2 : 1 in an aqueous medium as follows: FeCl_3_·6H_2_O (10 mmol) and FeCl_2_·4H_2_O (5 mmol) salts were completely dissolved in 100 mL deionized water. Then, the reaction mixture was transferred to a glass reactor equipped with a mechanical stirrer and a water bath preset at 80 °C. Under the N_2_ atmosphere, a sufficient amount of aqueous ammonia solution (28% w/w, 30 mL) was added to the stirring mixture to increase the reaction pH to 11. After that, a black dispersion was observed that vigorously stirred (800 rpm) for 1 h at room temperature and then refluxed for 1 h. The resulting nanoparticles were separated by the use of an external magnet from the aqueous solution, washed with distilled water and ethanol several times, and finally dried in an oven at 60 °C.

### Preparation of BHP@Fe_3_O_4_ organocatalyst

To prepare the final magnetic organocatalyst, 1 g of synthesized Fe_3_O_4_ nanoparticles was fully dispersed in a round-bottom flask of distilled water/MeOH (1 : 1 v/v) by the use of a bath ultrasonic for minutes. The BHP was completely dispersed in deionized water/MeOH by ultrasonic treatment for 20 min and then added to magnetic nanoparticle dispersion. Afterwards, the obtained mixture was stirred in a flask under an Ar atmosphere for at least 10 h. Finally, the prepared magnetically modified organocatalyst was separated and washed with deionized water/MeOH and then dried in an oven at 60 °C. After cooling to room temperature, the obtained fine powder was kept away from moisture before use in the transamidation reaction.

### The catalytic performance of BHP@Fe_3_O_4_ in transamidation reaction

**Scheme 3:**
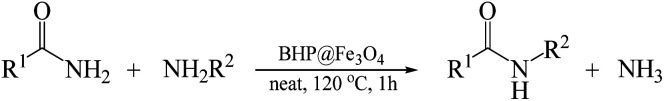
The model transamidation reaction.

Following a general procedure in Scheme 3, the amide (1 mmol), amine (1.5 mmol), and BHP@Fe_3_O_4_ magnetic catalyst were loaded in a 10 mL flask and the mixture was thoroughly stirred under an argon atmosphere at 120 °C for 2 h. The progress of the transamidation reaction was monitored by TLC. After completion of the reaction, the obtained mixture was firstly diluted with ethyl acetate and then the magnetic organocatalyst was collected from the reaction mixture by the use of an external magnet while it was washed repeatedly with an adequate amount of ethyl acetate to utilize in other cycles. The resulted reaction residue was concentrated under vacuum and purified by column chromatography to afford the desired product. Lastly, the structures of all pure products were identified *via* melting point, FTIR, ^1^H-NMR, ^13^C-NMR, and MS spectroscopies (ESI†).

## Conclusions

To sum up, BHP as a low-cost and abundant waste were immobilized by Fe_3_O_4_ nanoparticles *via* a simple method to estimate its catalytic performance in the synthesis of different amides derivatives through the transamidation reaction. The results indicated the yields at a good level under neat conditions. This phenomenon was expected because of presenting a large number of amino acids in the bovine horn structure as the keratin resource. Subsequently, the catalytic effectiveness not only remained with the corporation of magnetic nanoparticles but also facilitated the application of organocatalyst in the reusability aspect. While structural identification analysis demonstrated the successful synthesis of catalyst, TGA results showed the thermal stability of prepared catalyst even higher than the reaction temperature. So, our catalyst possesses added merits in terms of inexpensive, nontoxicity, easy synthesis as well as reusability. Since BHP is environmentally friendly, versatile plus high active to design many practical new catalysts for different reactions, we hope this work can be a motivation among researchers to use such wealth material even in practical industrial applications.

## Conflicts of interest

There are no conflicts to declare.

## Supplementary Material

RA-012-D1RA09327D-s001
