# Supplementary material for: Innovative application of magnetically modified bovine horn as a natural keratin resource in the role of value-added organocatalyst
Source: RSC Adv. 2022 Jun 6;12(26):16535–43. doi: 10.1039/d1ra09327d (PMC9169071; doi:10.1039/d1ra09327d)
Supplement: RA-012-D1RA09327D-s001 [file RA-012-D1RA09327D-s001.pdf]

Electronic Supplementary Material (ESI) for RSC Advances.
This journal is © The Royal Society of Chemistry 2022

Supplementary Information

for

Innovative Application of Magnetically Modified Bovine Horn as a Natural Keratin Resource in the Role of Value-Added Organocatalyst

Atefeh Darvishi[a], Hadi Bakhshi[b], Akbar Heydari[a,*]

[a] Chemistry Department, Tarbiat Modares University, Tehran, Iran. P.O. Box 14155-4838.
[b] Department of Life Science and Bioprocesses, Fraunhofer Institute for Applied Polymer Research IAP, Geiselbergstraße 69, 14476 Potsdam, Germany.
* Corresponding author, E-mail: heydar_a@modares.ac.ir

Table of Content

Content	Page
Spectra information of transamidation reaction derivatives	1-4
FTIR, ^{1}H-NMR, ^{13}C-NMR and MS spectra of amide derivatives	5-26

Spectra information of transamidation reaction derivatives

***N*-phenylacetamide (1c)**: The product was obtained as white solid in 90% isolated yield; Melting point: 110-112°C; IR (KBr) ν (cm^{-1}) = 3293, 2924, 2855, 1662, 1603, 1549, 1495, 1433, 1317, 1259, 753; ^{1}H-NMR: (500 MHz, DMSO-d_6, 25°C, TMS): δ (ppm) = 9.92 (s, 1H), 7.57 (d, *J* = 7.5 Hz, 2H), 7.27 (t, *J* = 7.5 Hz, 2H), 7.00 (t, *J* = 7.5 Hz, 1H), 2.03 (s, 3H).

***N-p*-tolylacetamide (2c)**: The product was obtained as white solid in 84% isolated yield; Melting point: 142-144°C; IR (KBr) ν (cm^{-1}) = 3296, 2930, 2863, 1663, 1607, 820; ^{1}H-NMR: (500 MHz, $CDCl_3$, 25°C, TMS): δ (ppm) = 7.37 (d, *J* = 8.25 Hz, 2H), 7.26 (s, 1H), 7.1 (d, *J* = 8.05 Hz, 2H), 2.3 (s, 3H), 2.15 (s, 3H); ^{13}C-NMR (125 MHz, $CDCl_3$, 25°C, TMS): δ (ppm) = 168.32, 135.33, 133.93, 129.52, 120.06, 24.46, 20.83.

***N*-(2-mercaptophenyl)acetamide (3c)**: The product was obtained as yellow solid in 76% isolated yield; Melting point: 150-152°C; IR (KBr): ν (cm^{-1}) = 3275, 2925, 1664, 1575, 1515, 1431, 1290, 752; ^{1}H-NMR (500 MHz, $CDCl_3$, 25°C, TMS): δ (ppm) = 8.28 (d, *J* = 7.45 Hz,1H), 8.05 (s, 1H), 7.62 (m,1H), 7.41 (m, 1H), 7.31 (m,1H), 7.07 (m, 1H), 2.25 (s, 3H).

***N*-(4-bromophenyl)acetamide (4c)**: The product was obtained as white solid in 92% isolated yield; Melting point: 163-164°C; IR (KBr): ν (cm^{-1}) = 3294, 2923, 2854,1666, 1596, 1003, 920; MS (EI, 70 eV): m/z (%) = 213 (M^+, 77), 178 (100), 145 (12), 143 (12), 119 (6), 92 (67), 91 (36), 65 (46).

***N*-benzylacetamide (5c)**: The product was obtained as white solid in 95% isolated yield; Melting point: 58-60 °C; IR (KBr): ν (cm^{-1}) = 3291, 3084, 2926, 2855, 1641, 1550, 1442, 1366, 1287, 1080, 1025, 742, 693; ^{1}H-NMR (400 MHz, $CDCl_3$, 25°C, TMS): δ (ppm) = 7.30-7.37 (m, 5H), 5.83 (br s, 1H), 4.45 (s, 2H), 2.05 (s, 3H).

***N*-benzylbenzamide (6c)**: The product was obtained as white solid in 96% isolated yield; Melting point: 100-102°C; IR (KBr): ν (cm^{-1}) = 3293, 2928, 2861, 1641, 1551, 1319, 594; ^{1}H-NMR (500 MHz, $CDCl_3$, 25°C, TMS): δ (ppm) = 9.07 (t, *J* = 5 Hz, 1H), 7.92 (m, 2H), 7.54 (m, 1H), 7.48 (m, 2H), 7.34 (m, 4H), 7.24 (m, 1H), 4.50 (d, *J* = 5 Hz, 2H); ^{13}C-NMR (125 MHz, $CDCl_3$, 25°C, TMS): δ (ppm) = 166.68, 140.18, 134.83, 131.70, 128.79, 127.72, 127.67, 127.19, 43.08.

***N*-(4-chlorobenzyl)benzamide (7c)**: The product was obtained as white solid in 78% isolated yield; IR (KBr) ν (cm^{-1}) = 3307, 3073, 2928, 1641, 1549, 1489, 1414, 1317, 1258, 1087, 990, 794, 692; ^{1}H-NMR (250 MHz, $CDCl_3$, 25°C, TMS): δ (ppm) = 7.72 (d, *J* = 8 Hz, 2H), 7.24-7.48 (m, 3H), 7.20 (s, 4H), 7.09 (s, 1H), 4.56 (d, *J* = 5.75 Hz, 2H).

***N*-(pyridine-3-ylmethyl)benzamide (8c)**: The product was obtained as white solid in 75% isolated yield; Melting point: 153-154°C; IR (KBr): ν (cm^{-1}) = 3443, 2927, 2859, 1753, 1643, 1539, 1460, 1373, 1264, 1023, 803; ^{1}H-NMR (500 MHz, $CDCl_3$, 25°C, TMS): δ (ppm) = 8.64 (s,

1H), 8.54 (s, 1H), 7.85 (d, *J* = 7.9 Hz, 2H), 7.81 (d, *J* = 7.3 Hz, 1H), 7.51 (t, *J* = 7.45 Hz,1H), 7.43 (t, *J* = 7.70 Hz, 2H), 7.32 (dd, *J* = 7.65, 5.05 Hz ,1H), 6.70 (s, 1H), 4.64 (d, *J* = 6.1Hz, 2H).

***N*-benzyl-4-methylbenzamide (9c)**: The product was obtained as white solid in 90% isolated yield; Melting point: 148-150°C; IR (KBr): ν (cm^{-1}) = 3310, 3065, 3056, 3025, 2916, 1640, 1547, 1420, 1360, 1323, 1257, 1057, 991, 842; ^{1}H-NMR (500 MHz, $CDCl_3$, 25°C, TMS): δ (ppm) = 9.01 (t, *J* = 5 Hz, 1 H), 7.84 (m, 2H), 7.29 (m, 7H), 4.49 (d, *J* = 5 Hz, 2H), 2.35 (s, 3H); ^{13}C-NMR (125 MHz, $CDCl_3$, 25°C, TMS): δ (ppm) = 166.58, 141.55, 140.30, 132.06, 129.30, 128.72, 127.76, 127.68, 127.15, 43.04, 21.41.

***N*-benzyl-4-chlorobenzamide (10c)**: The product was obtained as white solid in 87% isolated yield; Melting point: 157-160°C; IR (KBr): ν (cm^{-1}) = 3313, 3083, 3030, 1630, 1594, 1553, 1486, 1420, 1319, 1092, 849; ^{1}H-NMR (500 MHz, $CDCl_3$, 25°C, TMS): δ (ppm) = 9.15 (t, *J* = 5 Hz, 1H), 7.94 (d, *J* = 10 Hz, 2H), 7.55 (d, *J* = 5 Hz, 2H), 7.33 (d, *J* = 5Hz, 4H), 7.25 (q, *J* = 5 Hz, 1H), 4.50 (d, *J* = 5 Hz, 2H); ^{13}C-NMR (125 MHz, $CDCl_3$, 25°C, TMS): δ (ppm) = 165.65, 139.96, 136.57, 133.55, 129.68, 128.87, 128.76, 127.72, 127.25, 43.18.

***N*-benzyl-2-chlorobenzamide (11c)**: The product was obtained as white solid 79% isolated yield; Melting point: 158-161°C; IR (KBr): ν (cm^{-1}) = 3254, 3087, 3031, 2920, 2862, 1635, 1594, 1555, 1429, 1362, 1256, 1049, 767; ^{1}H-NMR (500 MHz, $CDCl_3$, 25°C, TMS): δ (ppm) = 9.01 (t, *J* = 5 Hz, 1H), 7.42 (m, 9H), 4.49 (d, *J* = 5 Hz, 2H); ^{13}C-NMR (125 MHz, $CDCl_3$, 25°C, TMS): δ (ppm) = 166.91, 139.63, 137.41, 131.23, 130.39, 130.10, 129.35, 128.78, 127.68, 127.58, 127.30, 42.95.

***N*-benzyl-4-nitrobenzamide (12c)**: The product was obtained as white solid 79% isolated yield; Melting point: 190-193°C; IR (KBr): ν (cm^{-1}) = 3430, 2252, 2125, 1619, 1584, 1341, 1026, 824, 762.^{1}H-NMR (500 MHz, $CDCl_3$, 25°C, TMS): δ (ppm) = 9.15 (t, *J* = 5 Hz, 1H), 7.94 (d, *J* = 10 Hz, 2H), 7.55 (d, *J* = 10 Hz, 2H), 7.33 (d, *J* = 5 Hz, 4H), 7.25 (q, *J* = 5 Hz, 1H), 4.50 (d, *J* = 5 Hz, 2H); ^{13}C-NMR (125 MHz, $CDCl_3$, 25°C, TMS): δ (ppm) = 168.41, 148.48, 145.89, 135.69, 130.47, 129.20, 128.94, 128.51, 123.22, 42.76.

***N*-propylbenzamide (13c)**: The product was obtained as white solid 91% isolated yield; Melting point: 105-107°C; IR (KBr): ν (cm^{-1}) = 3302, 3083, 2956, 2932, 2871, 1633, 1549, 1492, 1327, 1289, 1028, 803, 598; ^{1}H-NMR (500 MHz, $CDCl_3$, 25°C, TMS): δ (ppm) = 8.45 (t, *J* = 5 Hz, 1H), 7.85 (d, *J* = 10 Hz, 2H), 7.48 (m, 3H), 3.23 (m, 2H), 1.54 (m, 2H), 0.89 (m, 3H) ; ^{13}C-NMR (125 MHz, $CDCl_3$, 25°C, TMS): δ (ppm) = 166.6, 135.2, 131.4, 128.6, 127.5, 41.4, 22.9, 11.9.

Phenyl(piperidin-1-yl)methanone (14c): The product was obtained 70% isolated yield; IR (KBr): ν (cm^{-1}) = 3059, 2931, 2858, 1625, 1565, 1433, 1277; ^{1}H-NMR (250 MHz, $CDCl_3$, 25°C, TMS): δ (ppm) = 7.39 (s, 5H), 3.72 (s, 2H), 3.35 (s, 2H), 1.42-1.68 (m, 6H); ^{13}C-NMR (62.9 MHz, $CDCl_3$, 25°C, TMS): δ (ppm) = 170.4, 136.6, 129.5, 128.5, 126.9, 48.8, 43.2, 26.8, 25.8, 24.7; MS (EI, 70 eV): m/z (%) = 189 (M^+, 18), 160 (4), 133 (4), 111 (46), 105 (86), 84 (18), 77 (100).

4-benzoylmorpholin (15c): The product was obtained as colorless liquid 80% isolated yield; IR (KBr): ν (cm^{-1}) = 3296, 2956, 2927, 2867, 1637, 1535, 1454, 1048; ^{1}H-NMR: (250 MHz, $CDCl_3$, 25°C, TMS): δ (ppm) = 7.40 (s, 5H), 3.70 (br s, 2H), 3.46 (br s, 2H); ^{13}C-NMR (62.9 MHz, $CDCl_3$, 25°C, TMS): δ (ppm) = 170.6, 135.4, 130.0, 128.7, 127.2, 67.0, 48.2, 42.7; MS (EI, 70 eV): m/z (%) = 191 (M^+, 8), 176 (5), 148 (5), 114 (5), 105 (100), 86 (11), 77 (87).

***N*-(1-phenylethyl)benzamide (16c)**: The product was obtained as white solid 80% isolated yield; Melting point: 128-129°C; IR (KBr): ν (cm^{-1}) = 3329, 3030, 2967, 1635, 1525, 1449, 1080; ^{1}H-NMR (400 MHz, $CDCl_3$, 25°C, TMS): δ (ppm) = 7.79-7.81 (m, 2H), 7.30-7.54 (m, 8H), 6.34 (d, *J* = 5.2 Hz, 1H), 5.32-5.41 (m, 2H), 1.64 (d, *J* = 7.2 Hz, 3H); ^{13}C-NMR (100 MHz, $CDCl_3$, 25°C, TMS): δ (ppm) = 166.6, 143.1, 134.6, 131.5, 128.8, 128.6, 127.5, 126.9, 126.3, 49.2, 21.7; MS (EI, 70 eV): m/z (%) = 225 (M^+, 40), 210 (12), 120 (10), 105 (100), 91 (3), 77 (80).

4-methyl-*N*-(1-phenylethyl)benzamide (17c): The product was obtained as white solid 83% isolated yield; IR (KBr): ν (cm^{-1}) = 3331, 3032, 2973, 2925, 1632, 1522, 1450, 1270; ^{1}H-NMR (250 MHz, $CDCl_3$, 25°C, TMS): δ (ppm) = 7.77 (d, *J* = 7.7 Hz, 2H), 7.31-7.47 (m, 7H), 6.31 (br s, 1H), 5.31-5.37 (m, 1H), 2.41 (s, 3H), 1.61 (d, *J* = 6.7 Hz, 3H).

4-methoxy-*N*-(1-phenylethyl)benzamide (18c): The product was obtained as white solid 87% isolated yield; IR (KBr): ν (cm^{-1}) = 3434, 3337, 2818, 2849, 1622, 1526, 1502, 1257; ^{1}H-NMR (250 MHz, $CDCl_3$, 25°C, TMS): δ (ppm) = 7.74 (d, *J* = 8.7 Hz, 2H), 7.30-7.39 (m, 5H), 6.89-6.93 (m, 2H), 6.23 (br s, 1H), 5.31-5.36 (m, 1H), 3.84 (s, 3H), 1.60 (d, *J* = 6.7 Hz, 3H).

4-chloro-*N*-(1-phenylethyl)benzamide (19c): The product was obtained as white solid 78% isolated yield; IR (KBr): ν (cm^{-1}) = 3421, 3338, 2980, 1636, 1529, 1485, 1268; ^{1}H-NMR (250 MHz, $CDCl_3$, 25°C, TMS): δ (ppm) = 7.71 (d, *J* = 8.2 Hz, 2H), 7.30-7.40 (m, 7H), 6.39 (br s, 1H), 5.28-5.34 (m, 1H), 1.60 (d, *J* = 7.0, 3H).

***N*-phenylbenzamide (20c)**: The product was obtained as white solid 81% isolated yield; Melting point: 162-164°C; IR (KBr): ν (cm^{-1}) = 3343, 3051, 1655, 1599, 1532, 1437, 1321, 1259, 884, 750, 715, 590; ^{1}H-NMR (500 MHz, $CDCl_3$, 25°C, TMS): δ (ppm) = 9.30 (s, 1H), 7.8 (t, *J* = 10 Hz, 2H), 7.62 (d, *J* = 5 Hz, 2H), 7.37 (m, 1H), 7.30 (m, 2H), 7.19 (t, *J* = 5 Hz, 2H), 6.97 (t, *J* = 5 Hz, 1H); ^{13}C-NMR (125 MHz, $CDCl_3$, 25°C, TMS): δ (ppm) = 166.3, 138.7, 135.3, 131.4, 128.6, 128.3, 127.5, 124.0, 120.6.

4-methyl-*N*-phenylbenzamide (21c): The product was obtained as white solid 76% isolated yield; Melting point: 146-148°C; IR (KBr): ν (cm^{-1}) = 3351, 3057, 2916, 1649, 1596, 1524, 1438, 1320, 1260, 885, 747, 588; ^{1}H-NMR (500 MHz, $CDCl_3$, 25°C, TMS): δ (ppm) = 10.20 (s, 1H), 7.88 (m, 4H), 7.35 (m, 4H), 7.10 (m, 1H), 2.38 (s, 3H); ^{13}C-NMR (125 MHz, $CDCl_3$, 25°C, TMS): δ (ppm) = 165.8, 142.0, 139.7, 132.5, 129.3, 129.0, 128.2, 124.0, 120.8, 21.4.

4-chloro-*N*-phenylbenzamide (22c): The product was obtained as white solid 75% isolated yield; Melting point: 197-200°C; IR (KBr): ν (cm^{-1}) = 3351, 3083, 3056, 1653, 1597, 1528,

1438, 1324, 1258, 1092, 847, 755, 589; ^{1}H-NMR (500 MHz, $CDCl_3$, 25°C, TMS): δ (ppm) = 10.33 (s, 1H), 8.01 (m, 2H), 7.79 (m, 2H), 7.60 (m, 2H), 7.36 (m, 2H), 7.11 (m, 1H); ^{13}C-NMR (125 MHz, $CDCl_3$, 25°C, TMS): δ (ppm) = 164.9, 139.4, 136.9, 134.1, 130.1, 129.1, 128.9, 124.3, 120.9.

2-chloro-*N*-phenylbenzamide (23c): The product was obtained as white solid 71% isolated yield; Melting point: 150-151°C; IR (KBr): ν (cm^{-1}) = 3238, 3187, 3134, 3078, 2980, 1651, 1599, 1546, 1488, 1329, 1262, 1123, 1049, 768, 691; ^{1}H-NMR (500 MHz, $CDCl_3$, 25°C, TMS): δ (ppm) = 10.52 (s, 1H), 7.76 (d, *J* = 5 Hz, 2H), 7.59 (m, 2H), 7.50 (m, 2H), 7.37 (t, *J* = 5 Hz, 2H), 7.13 (t, *J* = 5 Hz, 1H) ; ^{13}C-NMR (125 MHz, $CDCl_3$, 25°C, TMS): δ (ppm) = 165.4, 139.4, 137.5, 131.5, 130.4, 130.1, 129.4, 129.2, 127.7, 124.3, 120.1.

4-nitro-*N*-phenylbenzamide (24c): The product was obtained as white solid 72% isolated yield; Melting point: 181-182°C; IR (KBr): ν (cm^{-1}) = 3321, 3110, 1621, 1592, 1523, 1407, 1351, 1317, 1262, 1105, 832, 724, 506.^{1}H-NMR (500 MHz, $CDCl_3$, 25°C, TMS): δ (ppm) = 10.93 (s, 1H), 8.33 (d, *J* = 10 Hz, 2H), 8.27 (d, *J* = 10 Hz, 2H), 7.85 (d, *J* = 10 Hz, 2H), 7.36 (t, *J* = 5 Hz, 2H), 7.13 (t, *J* = 5 Hz, 1H); ^{13}C-NMR (125 MHz, $CDCl_3$, 25°C, TMS): δ (ppm) = 168.47, 164.37, 149.5, 148.3, 146.7, 141.1, 139.3, 130.5, 129.8, 129.1, 124.5, 123.9, 123.1, 121.1.

1,3-diphenylurea (25c): The product was obtained as white solid 86% isolated yield; Melting point: 238-241°C; IR (KBr): ν (cm^{-1}) = 3323, 1645, 1599, 1549, 1439, 1306, 1230.

1,3-bis(1-phenylethyl)urea (26c): The product was obtained as white solid 90% isolated yield; IR (KBr): ν (cm^{-1}) = 3329, 2969, 1626, 1575, 1447, 1248, 1115; ^{1}H-NMR (400 MHz, $CDCl_3$, 25°C, TMS): δ (ppm) = 7.27-7.31 (m, 10H), 5.99 (br s, 2H), 4.79 (q, *J* =8.0 Hz, 2H), 1.43 (d, *J* =8.0 Hz, 6H).

1,3-diphenylthiourea (27c): The product was obtained as white solid 80% isolated yield; Melting point: 153-155°C; IR (KBr): ν (cm^{-1}) = 3204, 3020, 1545, 1449, 1339, 1234, 1068; ^{1}H-NMR (500 MHz, DMSO-d_6, 25°C, TMS): δ (ppm) = 9.79 (s, 2H), 7.47 (t, *J* = 7.5 Hz, 2H), 7.32 (t, *J* = 7.5 Hz, 2H), 7.11 (t, *J* = 7.5 Hz, 1H).

^{1}H-NMR, ^{13}C-NMR and mass spectra of amide derivatives

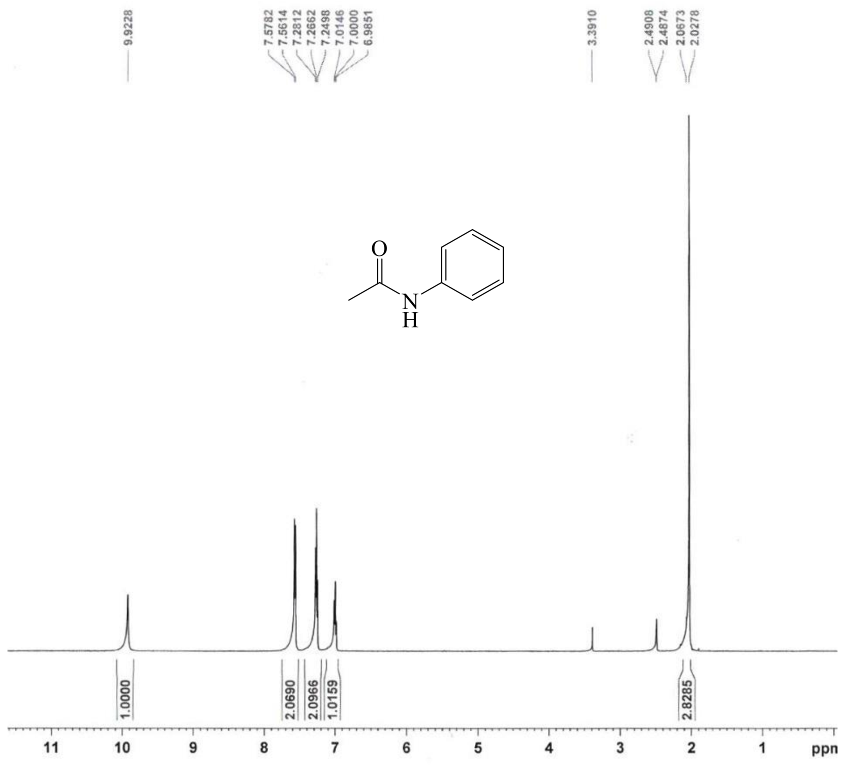

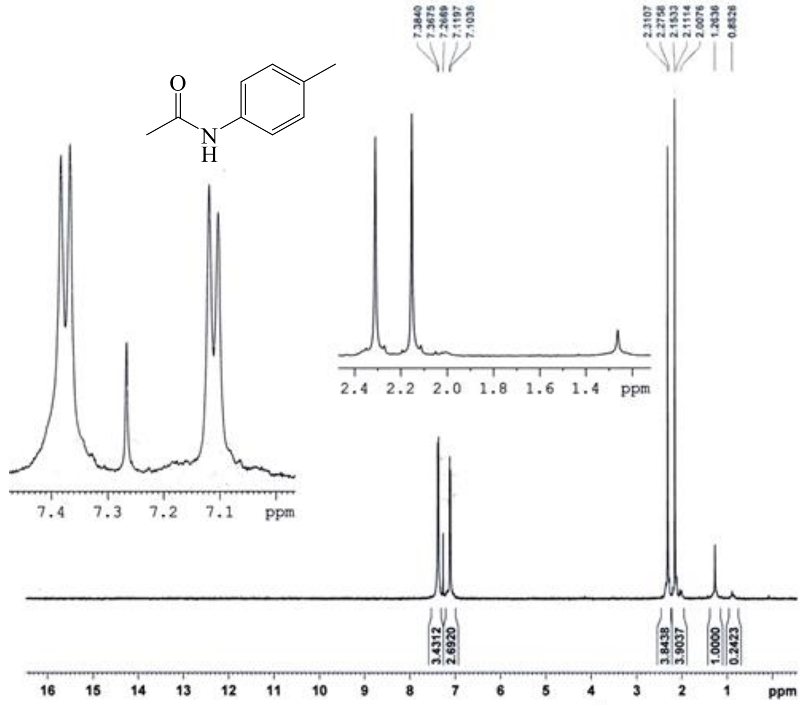

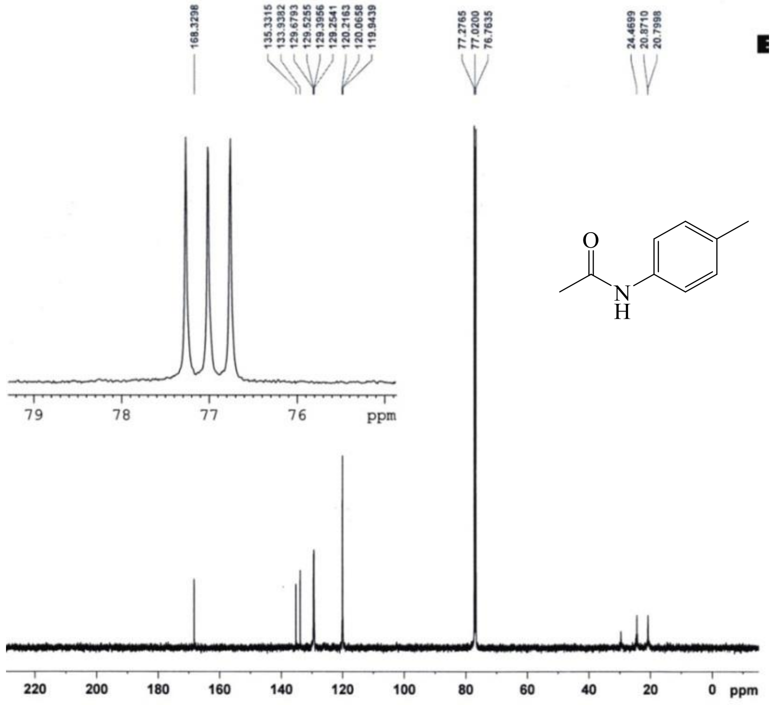
168.3298
135.3315
133.9382
129.6793
129.5255
129.3956
129.2541
120.2163
120.0658
119.9439
77.2765
77.0200
76.7635
24.4699
20.8710
20.7998
O
N
H
79 78 77 76 ppm
220 200 180 160 140 120 100 80 60 40 20 0 ppm

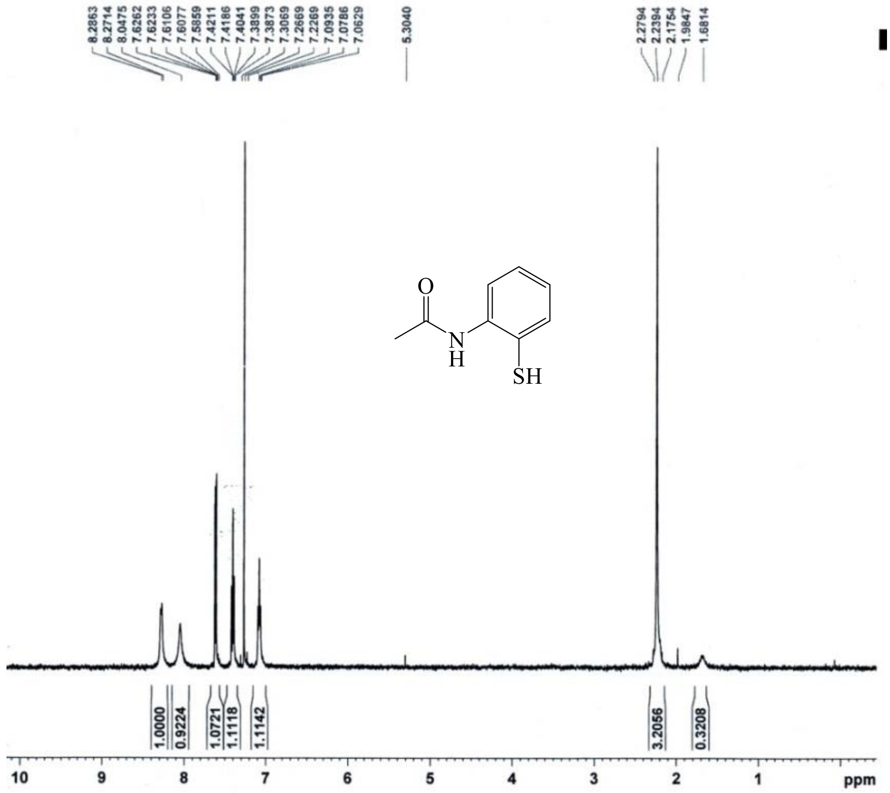
8.2863
8.2714
8.0475
7.6262
7.6233
7.6106
7.6077
7.5859
7.4211
7.4186
7.4041
7.3899
7.3873
7.3069
7.2669
7.2269
7.0935
7.0786
7.0629
5.3040
2.2794
2.2394
2.1754
1.9847
1.6814
O
N
H
SH
1.0000
0.9224
1.0721
1.1118
1.1142
3.2056
0.3208
10 9 8 7 6 5 4 3 2 1 ppm

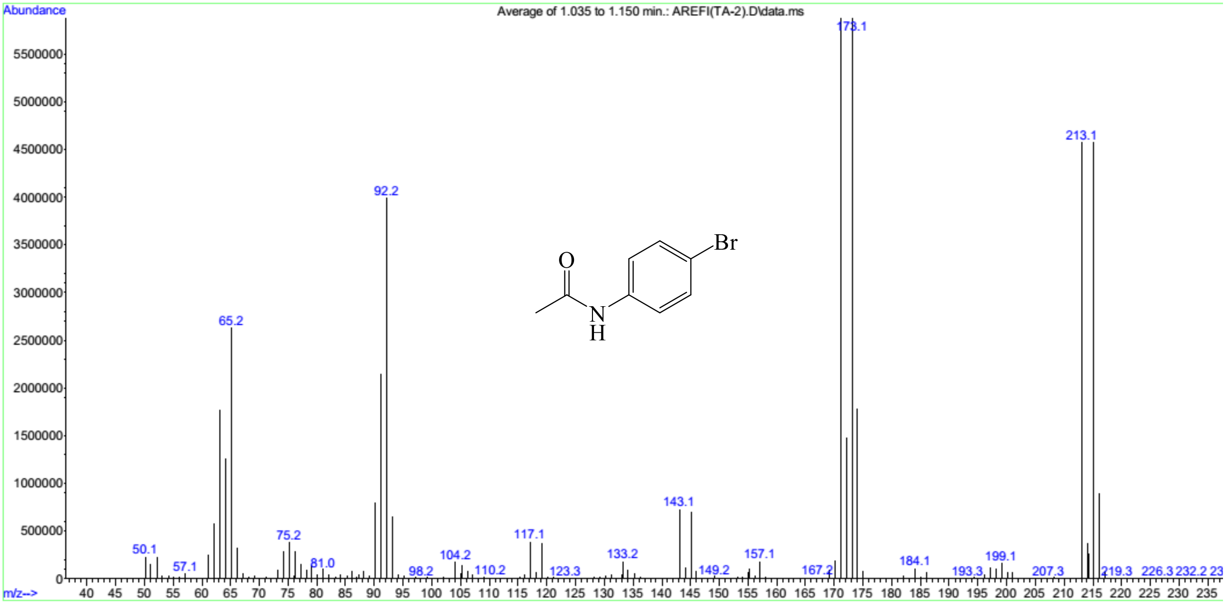
Abundance
Average of 1.035 to 1.150 min.: AREFI(TA-2).D\data.ms
m/z-->

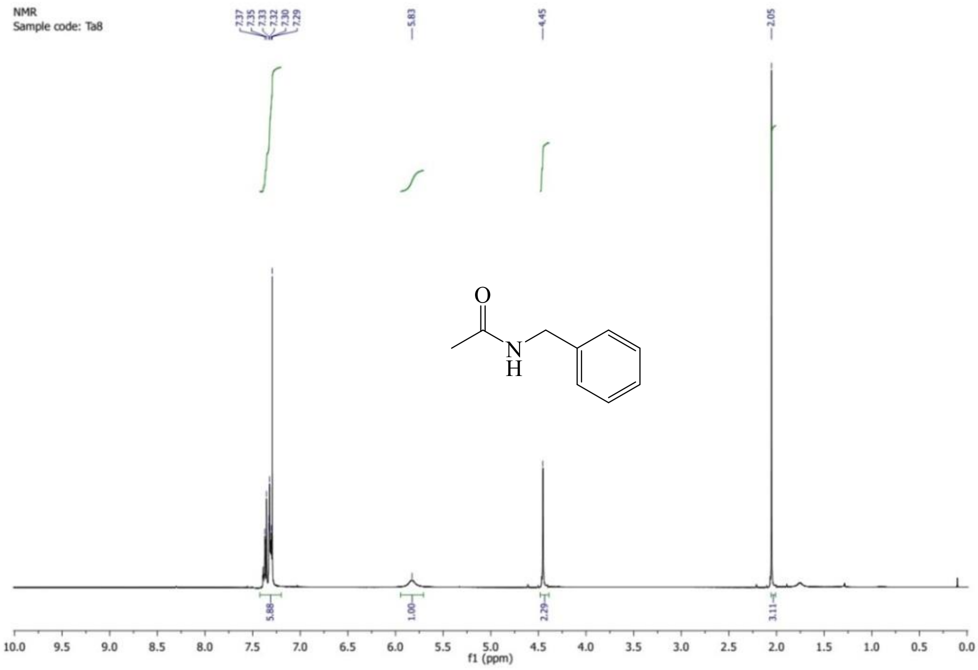
NMR
Sample code: Ta8
f1 (ppm)

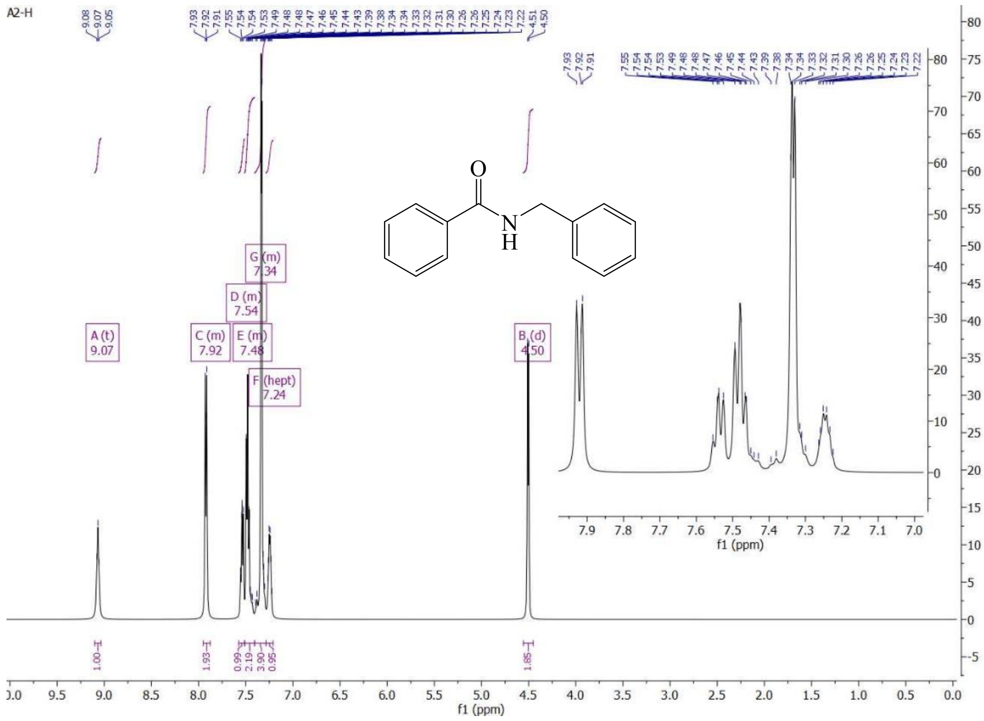
A2-H
9.08
9.07
9.05
4.51
4.50
A (t)
9.07
C (m)
7.92
D (m)
7.54
E (m)
7.48
G (m)
7.34
F (hept)
7.24
B (d)
4.50
1.00
1.93
0.99
2.19
3.90
0.95
1.85
O
N
H
f1 (ppm)

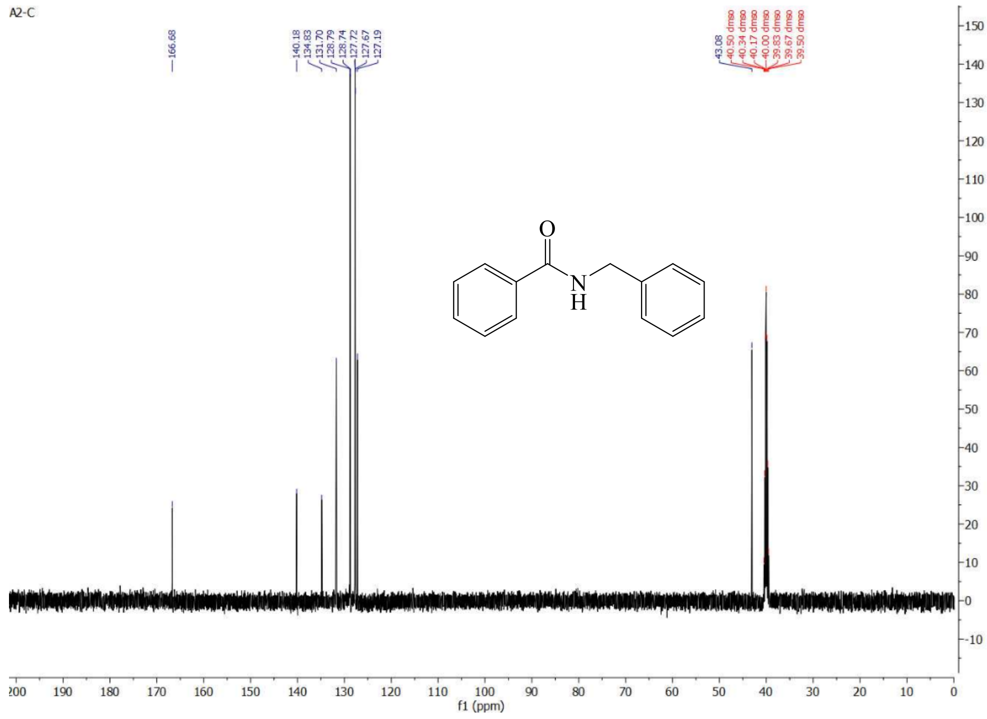
A2-C
166.68
140.18
134.83
131.70
128.79
128.74
127.72
127.67
127.19
43.08
40.50 dmso
40.34 dmso
40.17 dmso
40.00 dmso
39.83 dmso
39.67 dmso
39.50 dmso
O
N
H
f1 (ppm)

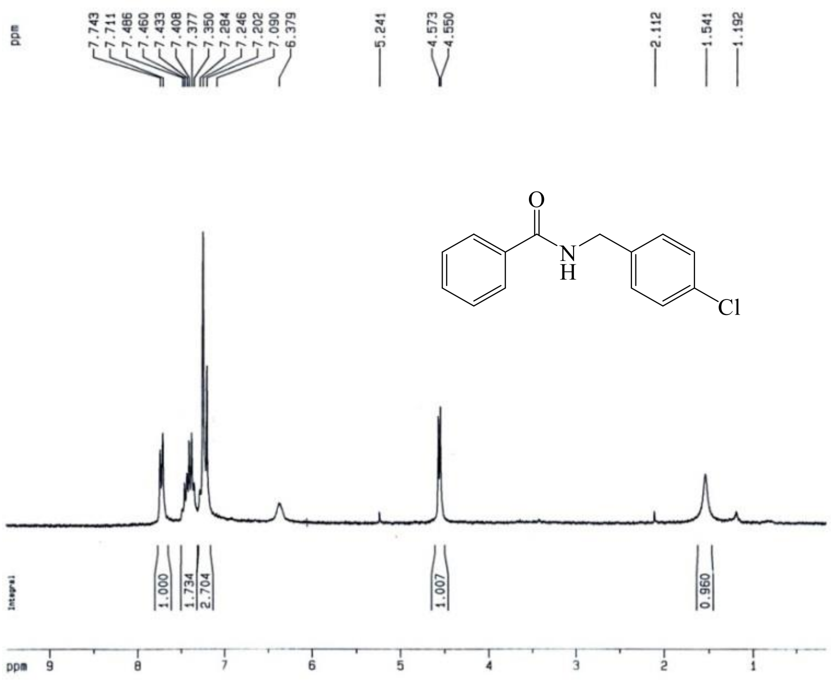
ppm
7.743
7.711
7.486
7.460
7.433
7.408
7.377
7.350
7.284
7.246
7.202
7.090
6.379
5.241
4.573
4.550
2.112
1.541
1.192
O
N
H
Cl
Integral
1.000
1.734
2.704
1.007
0.960
ppm 9 8 7 6 5 4 3 2 1

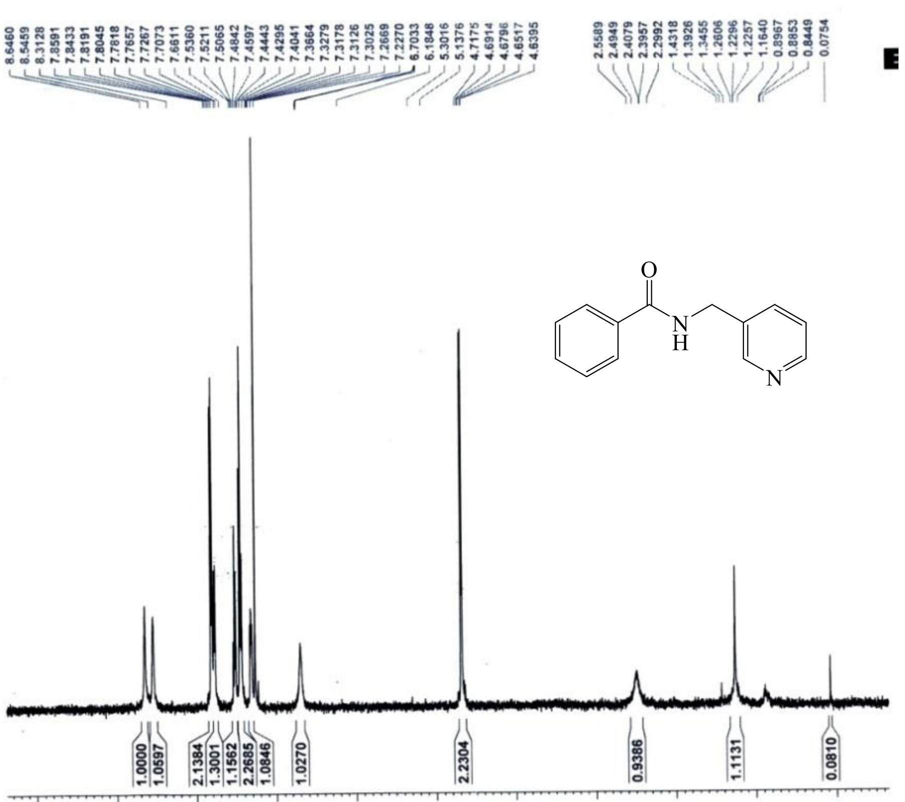
8.6460
8.5459
8.3128
7.8591
7.8433
7.8191
7.8045
7.7818
7.7657
7.7267
7.7073
7.6611
7.5360
7.5211
7.5065
7.4842
7.4597
7.4443
7.4295
7.4041
7.3664
7.3279
7.3178
7.3126
7.3025
7.2669
7.2270
6.7033
6.1848
5.3016
5.1376
4.7175
4.6914
4.6796
4.6517
4.6395
2.5589
2.4949
2.4079
2.3957
2.2992
1.4318
1.3926
1.3455
1.2606
1.2296
1.2257
1.1640
0.8967
0.8853
0.8449
0.0754
O
N
H
N
1.0000
1.0597
2.1384
1.3001
1.1562
2.2685
1.0846
1.0270
2.2304
0.9386
1.1131
0.0810
10 9 8 7 6 5 4 3 2 1 0 ppm

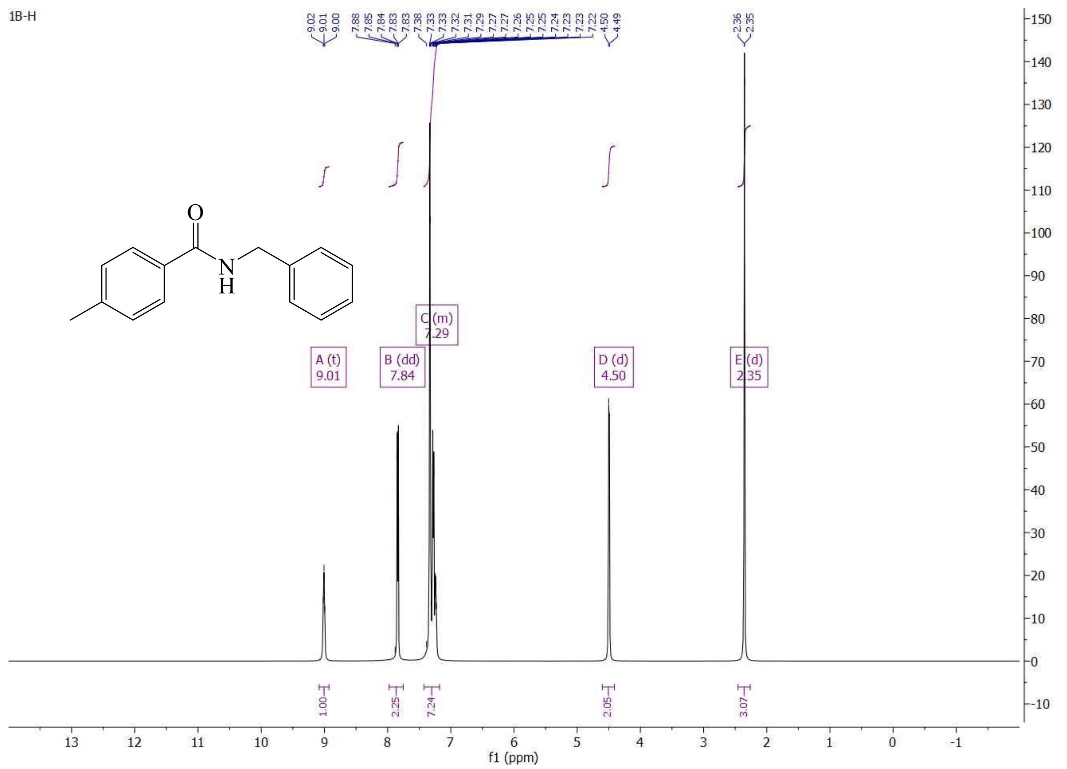
1B-H
9.02 9.01 9.00 7.88 7.85 7.84 7.83 7.83 7.38 7.33 7.33 7.32 7.31 7.29 7.27 7.27 7.26 7.25 7.25 7.24 7.23 7.23 7.22 4.50 4.49 2.36 2.35
O
N
H
A (t) 9.01
B (dd) 7.84
C (m) 7.29
D (d) 4.50
E (d) 2.35
1.00 2.25 7.24 2.05 3.07
f1 (ppm)

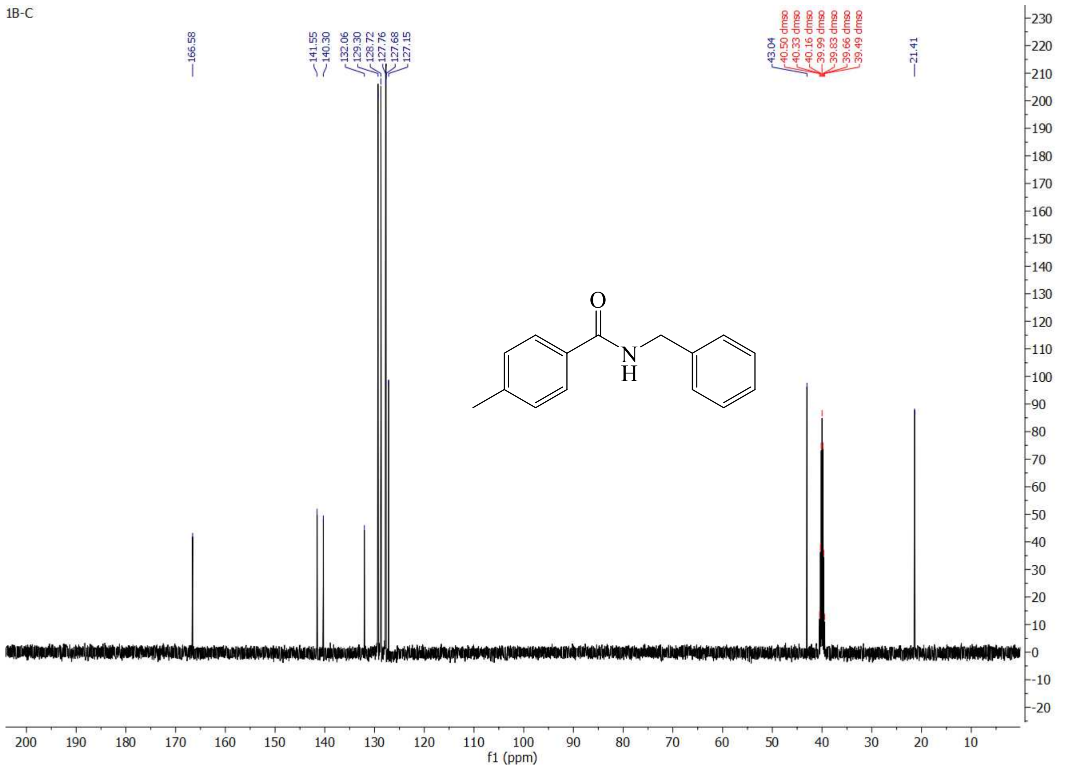
1B-C
166.58
141.55 140.30 132.06 129.30 128.72 127.76 127.68 127.15
43.04 40.50 dmso 40.33 dmso 40.16 dmso 39.99 dmso 39.83 dmso 39.66 dmso 39.49 dmso
21.41
O
N
H
f1 (ppm)

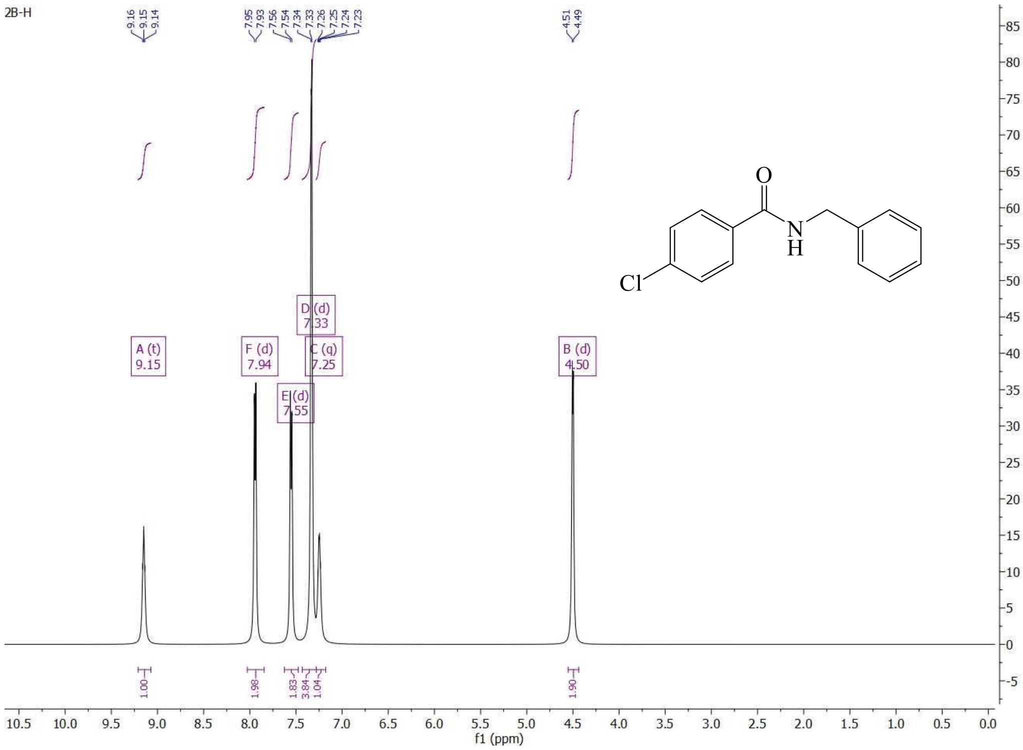
2B-H
9.16
9.15
9.14
7.95
7.93
7.56
7.54
7.34
7.33
7.26
7.25
7.24
7.23
4.51
4.49
A (t)
9.15
F (d)
7.94
D (d)
7.33
C (q)
7.25
E (d)
7.55
B (d)
4.50
1.00
1.98
1.83
3.84
1.04
1.90
O
N
H
Cl
f1 (ppm)

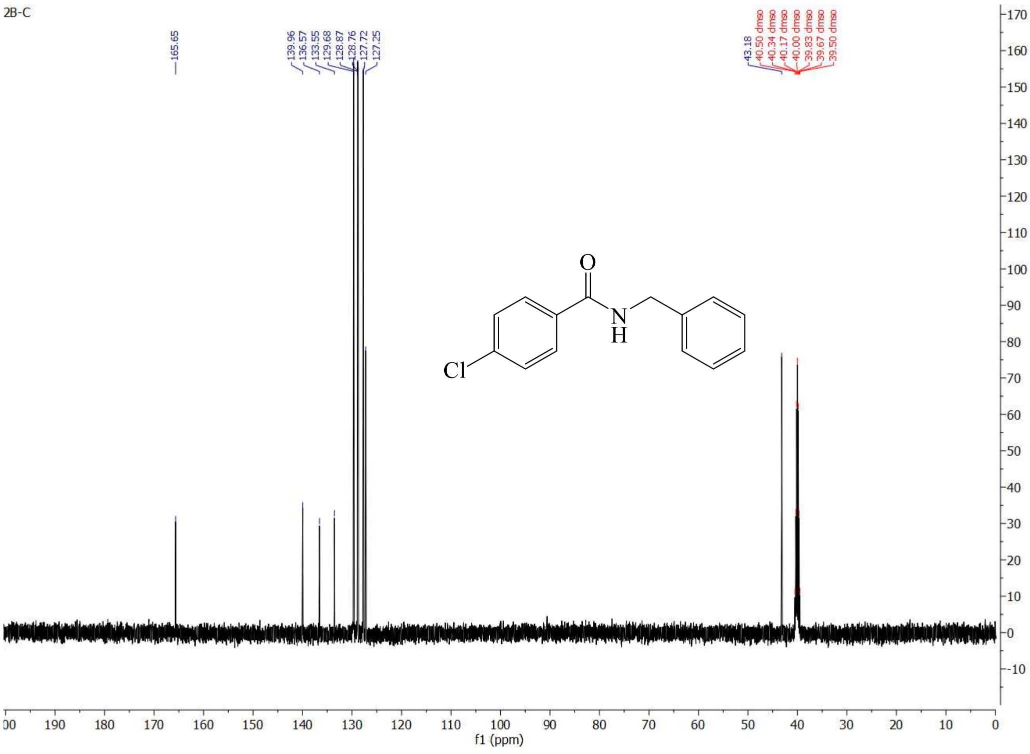
2B-C
165.65
139.96
136.57
133.55
129.68
128.87
128.76
127.72
127.25
43.18
40.50 dmso
40.34 dmso
40.17 dmso
40.00 dmso
39.83 dmso
39.67 dmso
39.50 dmso
O
N
H
Cl
f1 (ppm)

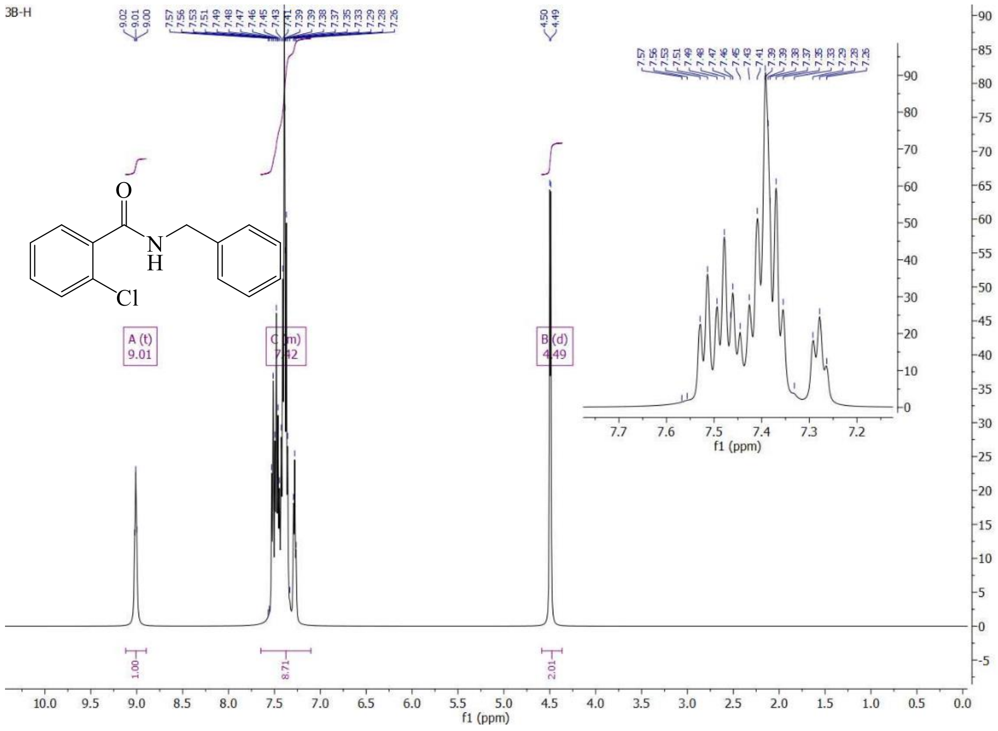
3B-H
A (t)
9.01
C (m)
7.42
B (d)
4.49
f1 (ppm)

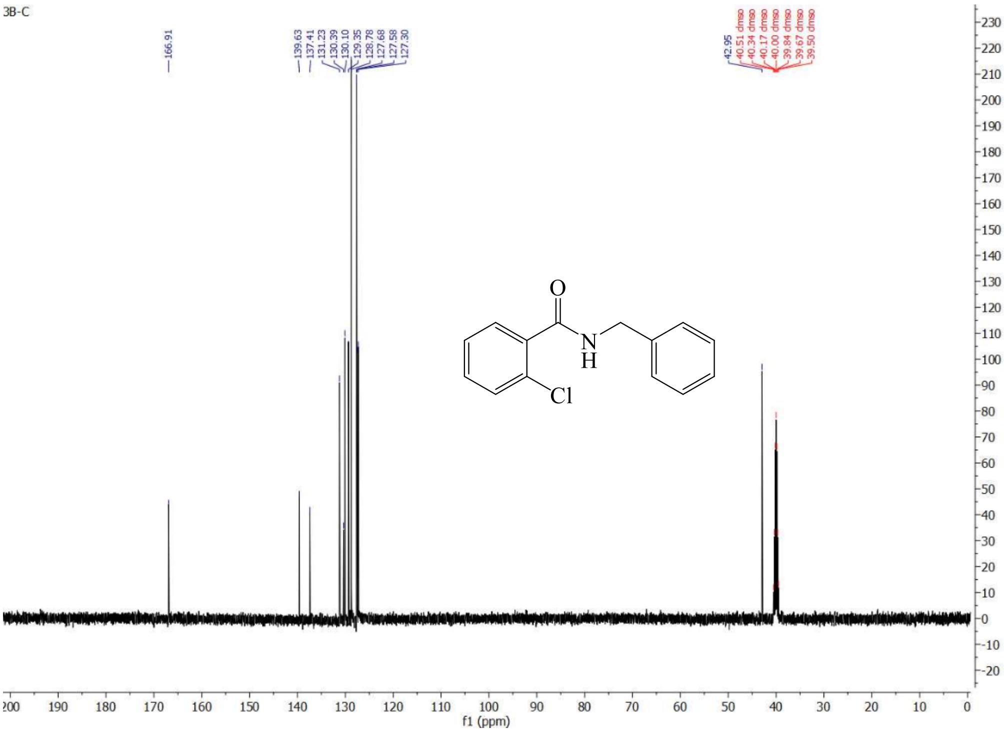
3B-C
166.91
f1 (ppm)

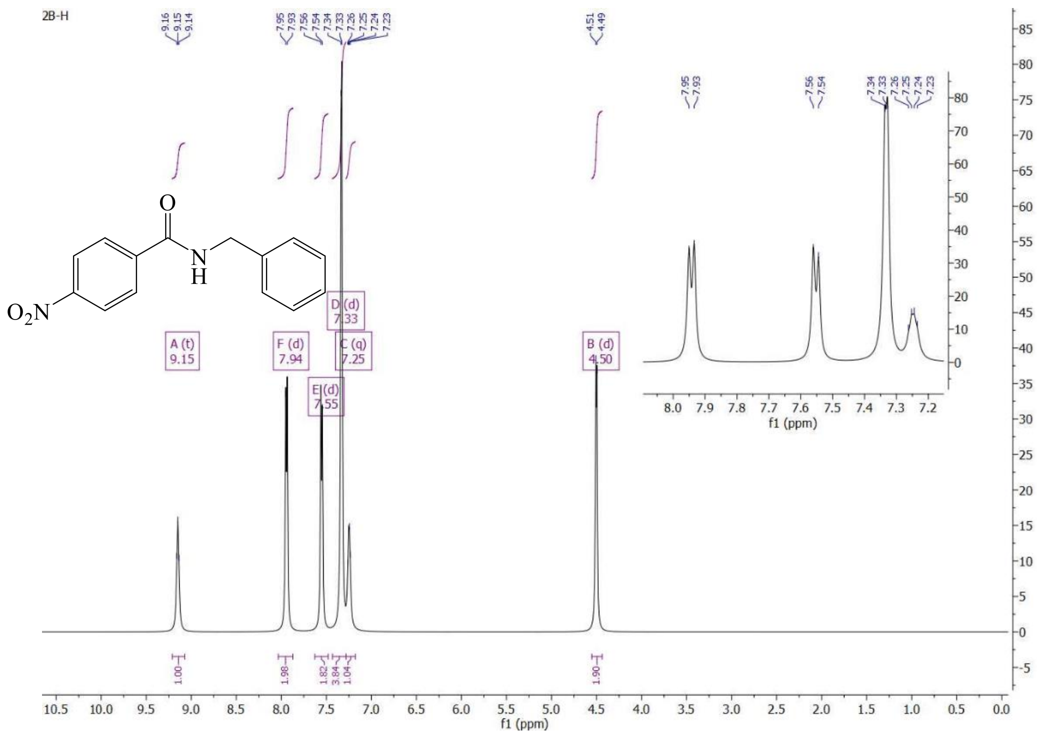
2B-H
A (t)
9.15
F (d)
7.94
E (d)
7.55
D (d)
7.33
C (q)
7.25
B (d)
4.50
f1 (ppm)

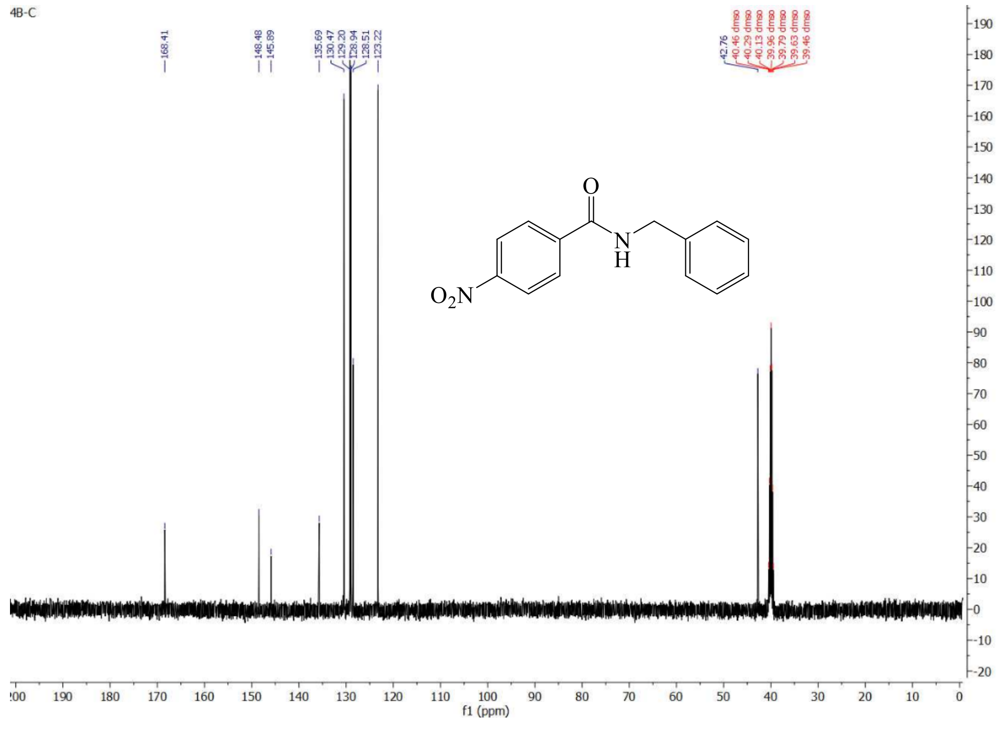
4B-C
168.41
148.48
145.89
135.69
130.47
129.20
128.94
128.51
123.22
42.76
f1 (ppm)

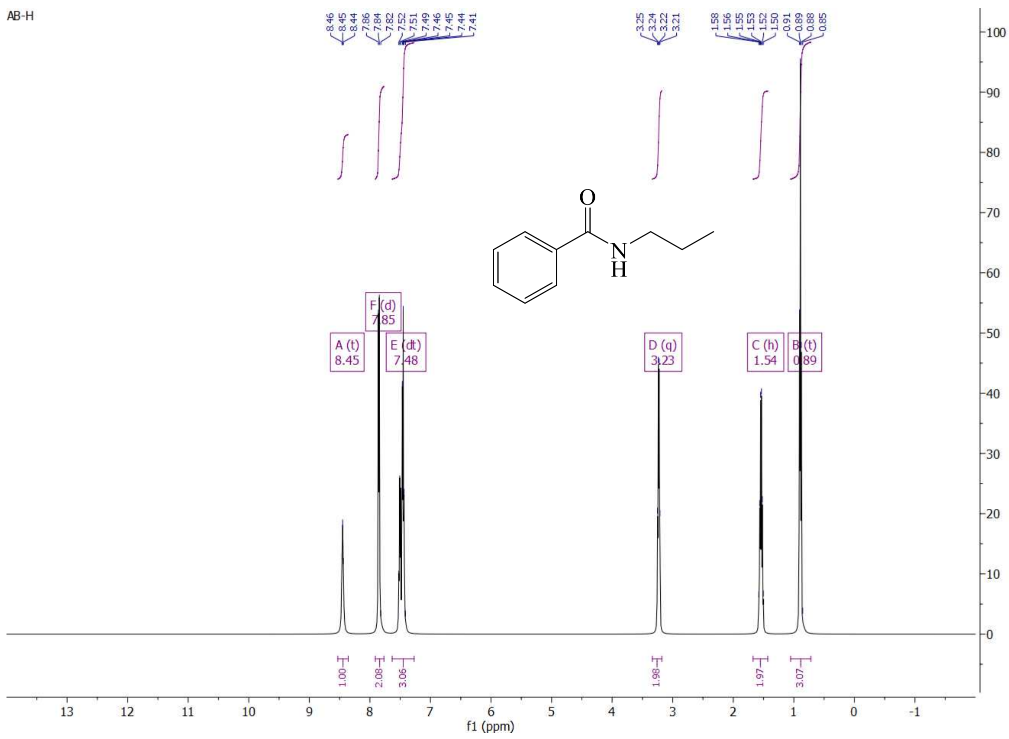
AB-H
8.46
8.45
8.44
7.86
7.84
7.82
7.52
7.51
7.49
7.46
7.45
7.44
7.41
3.25
3.24
3.22
3.21
1.58
1.56
1.55
1.53
1.52
1.50
0.91
0.89
0.88
0.85
O
N
H
F (d)
7.85
A (t)
8.45
E (dt)
7.48
D (q)
3.23
C (h)
1.54
B (t)
0.89
1.00
2.08
3.06
1.98
1.97
3.07
f1 (ppm)

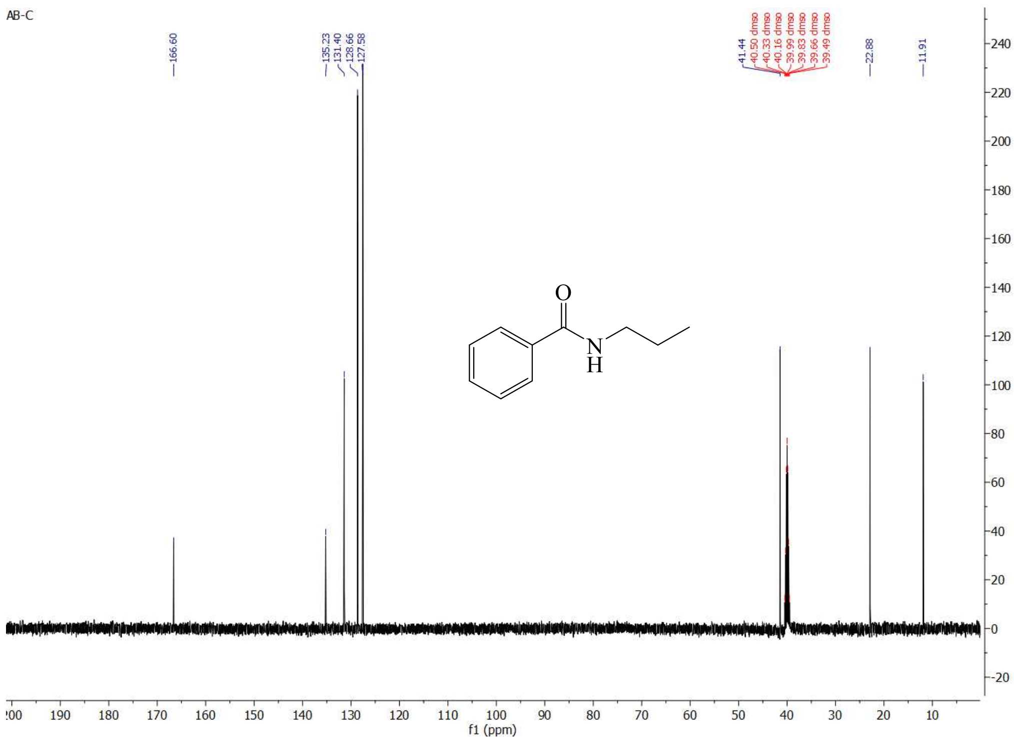
AB-C
166.60
135.23
131.40
128.66
127.58
41.44
40.50 dmso
40.33 dmso
40.16 dmso
39.99 dmso
39.83 dmso
39.66 dmso
39.49 dmso
22.88
11.91
O
N
H
f1 (ppm)

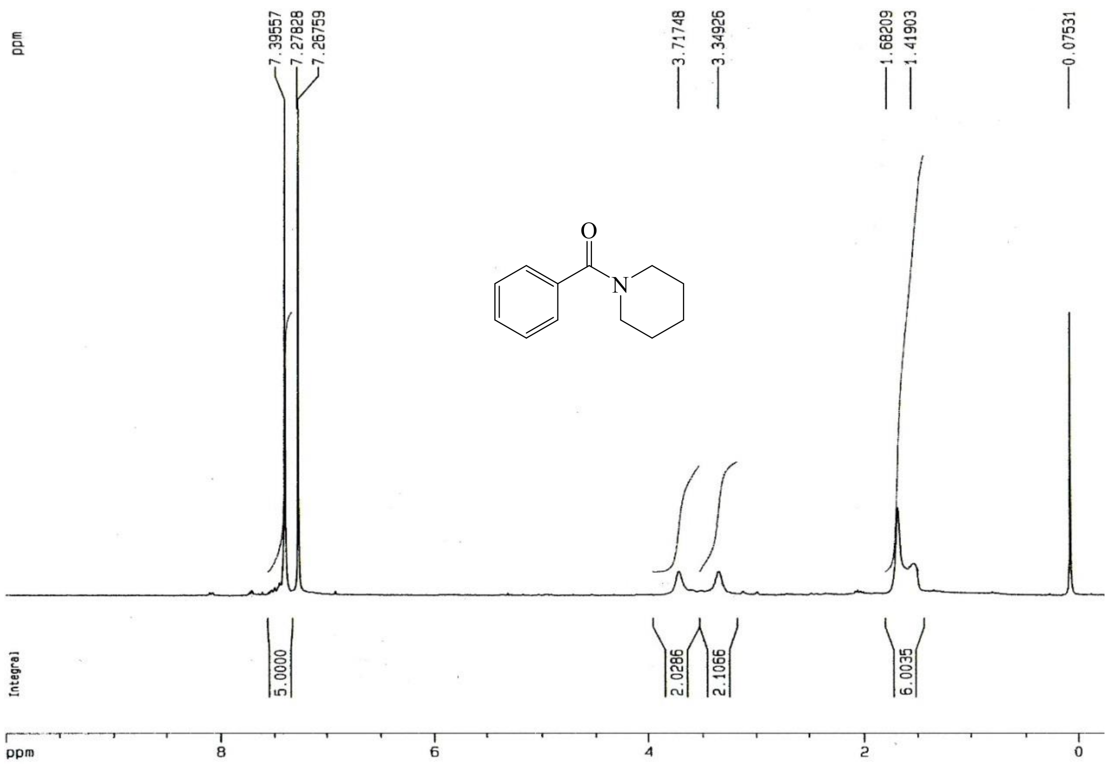
ppm
7.39557
7.27828
7.26759
3.71748
3.34926
1.68209
1.41903
0.07531
O
N
Integral
5.0000
2.0286
2.1066
6.0035
ppm
8
6
4
2
0

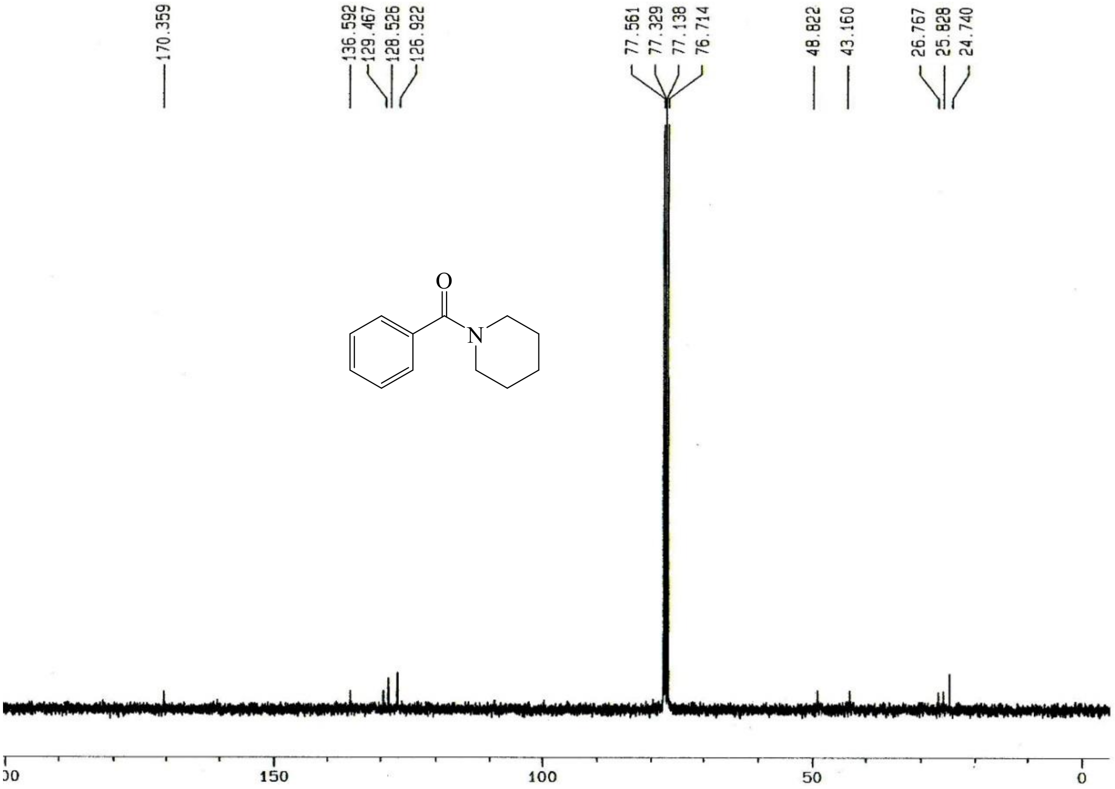
170.359
136.592
129.467
128.526
126.922
77.561
77.329
77.138
76.714
48.822
43.160
26.767
25.828
24.740
O
N
150
100
50
0

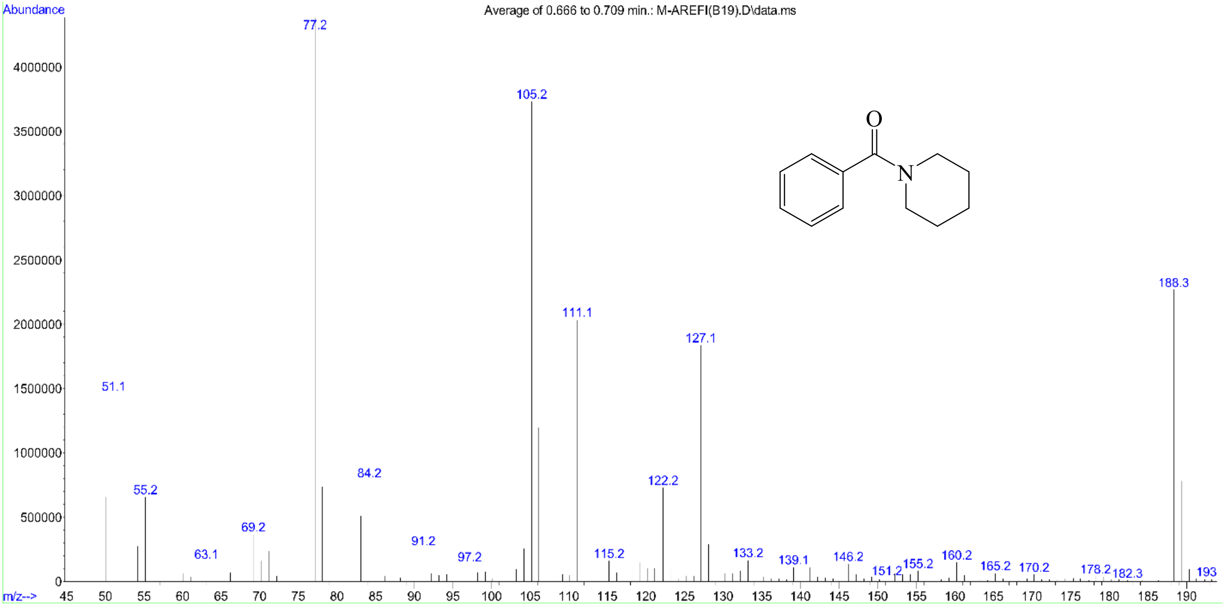
Abundance
Average of 0.666 to 0.709 min.: M-AREFI(B19).D\data.ms
m/z-->
77.2
105.2
111.1
127.1
188.3
51.1
55.2
63.1
69.2
84.2
91.2
97.2
115.2
122.2
133.2
139.1
146.2
151.2
155.2
160.2
165.2
170.2
178.2
182.3
193
O
N

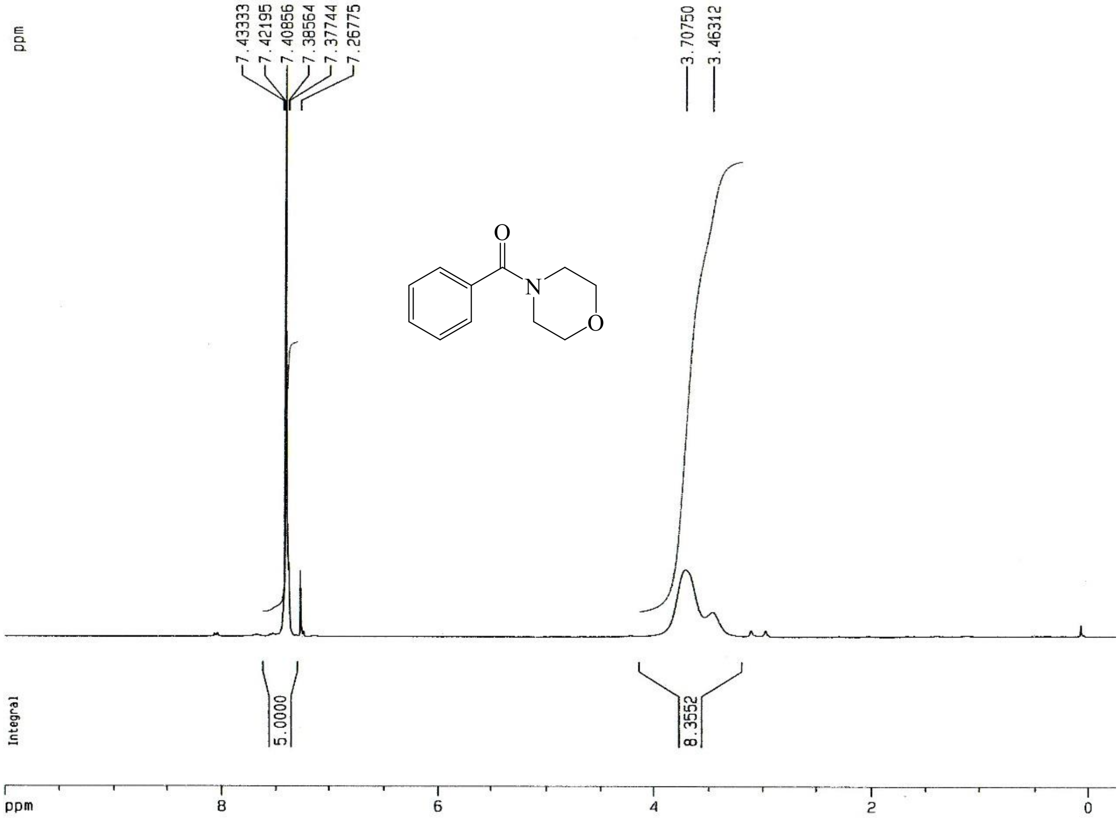
ppm
7.43333
7.42195
7.40856
7.38564
7.37744
7.26775
3.70750
3.46312
Integral
5.0000
8.3552
O
N
O

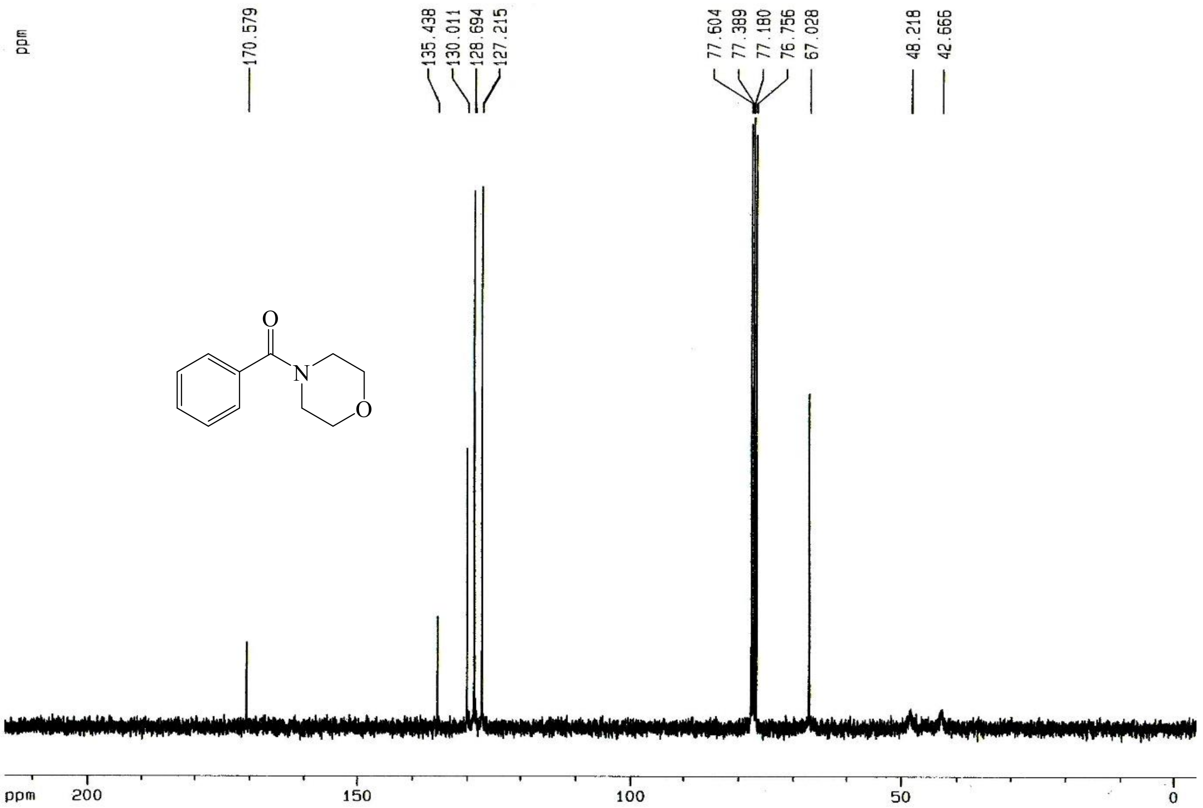
ppm
170.579
135.438
130.011
128.694
127.215
77.604
77.389
77.180
76.756
67.028
48.218
42.666
O
N
O
ppm 200 150 100 50 0

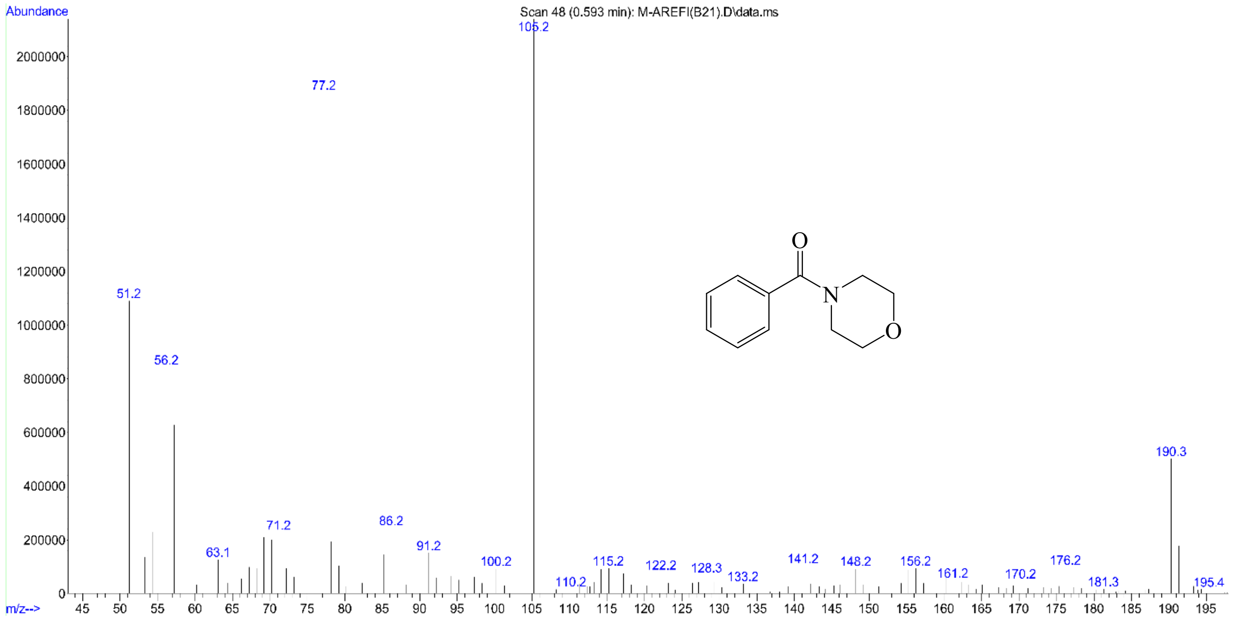
Abundance
Scan 48 (0.593 min): M-AREFI(B21).D\data.ms
105.2
77.2
51.2
56.2
63.1
71.2
86.2
91.2
100.2
110.2
115.2
122.2
128.3
133.2
141.2
148.2
156.2
161.2
170.2
176.2
181.3
190.3
195.4
m/z-->
O
N
O

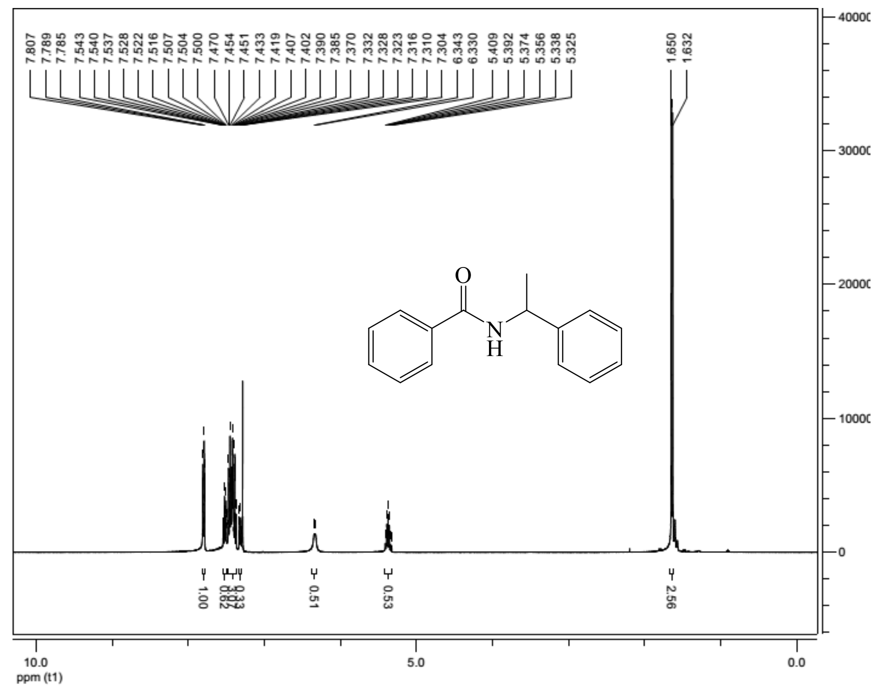
7.807
7.789
7.785
7.543
7.540
7.537
7.528
7.522
7.516
7.507
7.504
7.500
7.470
7.454
7.451
7.433
7.419
7.407
7.402
7.390
7.385
7.370
7.332
7.328
7.323
7.316
7.310
7.304
6.343
6.330
5.409
5.392
5.374
5.356
5.338
5.325
1.650
1.632
O
N
H
1.00
0.51
0.53
2.56
10.0
ppm (t1)
5.0
0.0
40000
30000
20000
10000
0

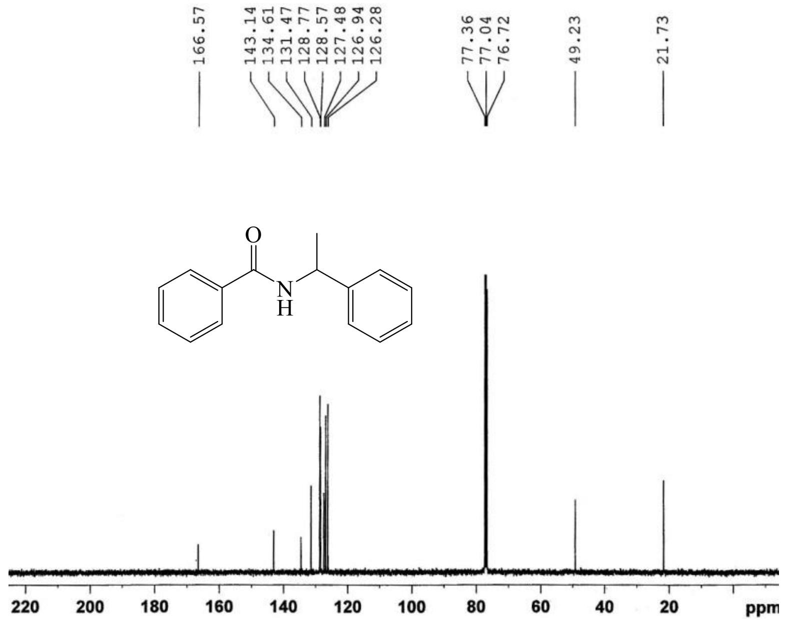
166.57
143.14
134.61
131.47
128.77
128.57
127.48
126.94
126.28
77.36
77.04
76.72
49.23
21.73
O
N
H
220
200
180
160
140
120
100
80
60
40
20
ppm

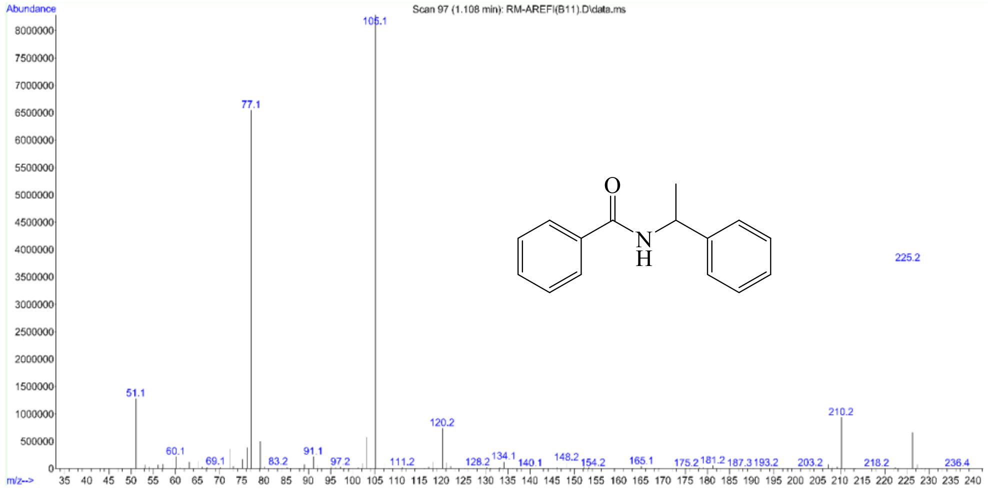
Abundance
Scan 97 (1.108 min): RM-AREFI(B11).D\data.ms
m/z-->
105.1
77.1
51.1
60.1
69.1
83.2
91.1
97.2
111.2
120.2
128.2
134.1
140.1
148.2
154.2
165.1
175.2
181.2
187.3
193.2
203.2
210.2
218.2
225.2
236.4
O
N
H

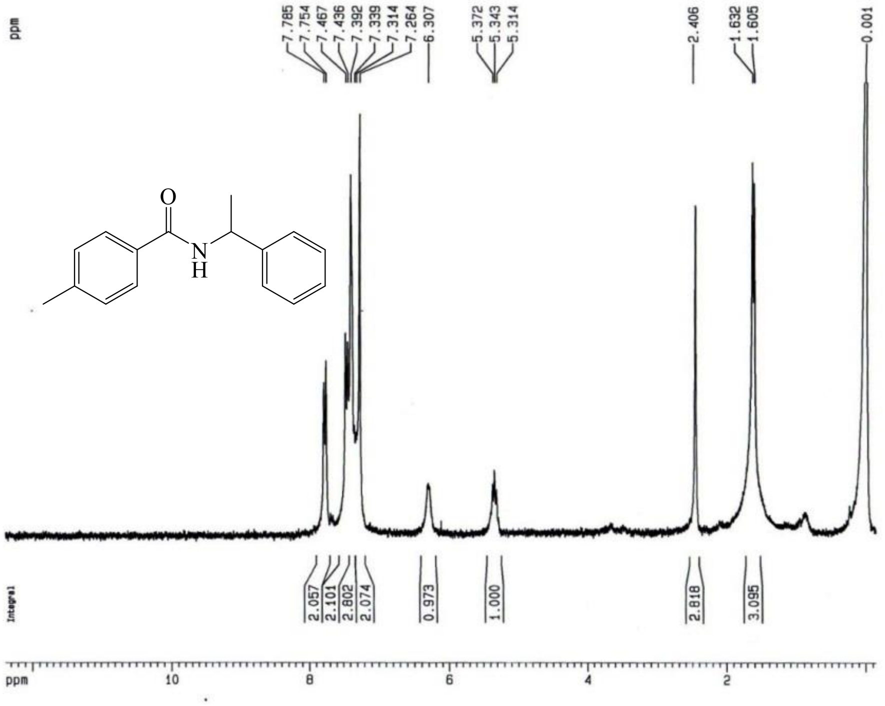
ppm
7.785
7.754
7.467
7.436
7.392
7.339
7.314
7.264
6.307
5.372
5.343
5.314
2.406
1.632
1.605
0.001
O
N
H
Integral
2.057
2.101
2.802
2.074
0.973
1.000
2.818
3.095
ppm
10
8
6
4
2

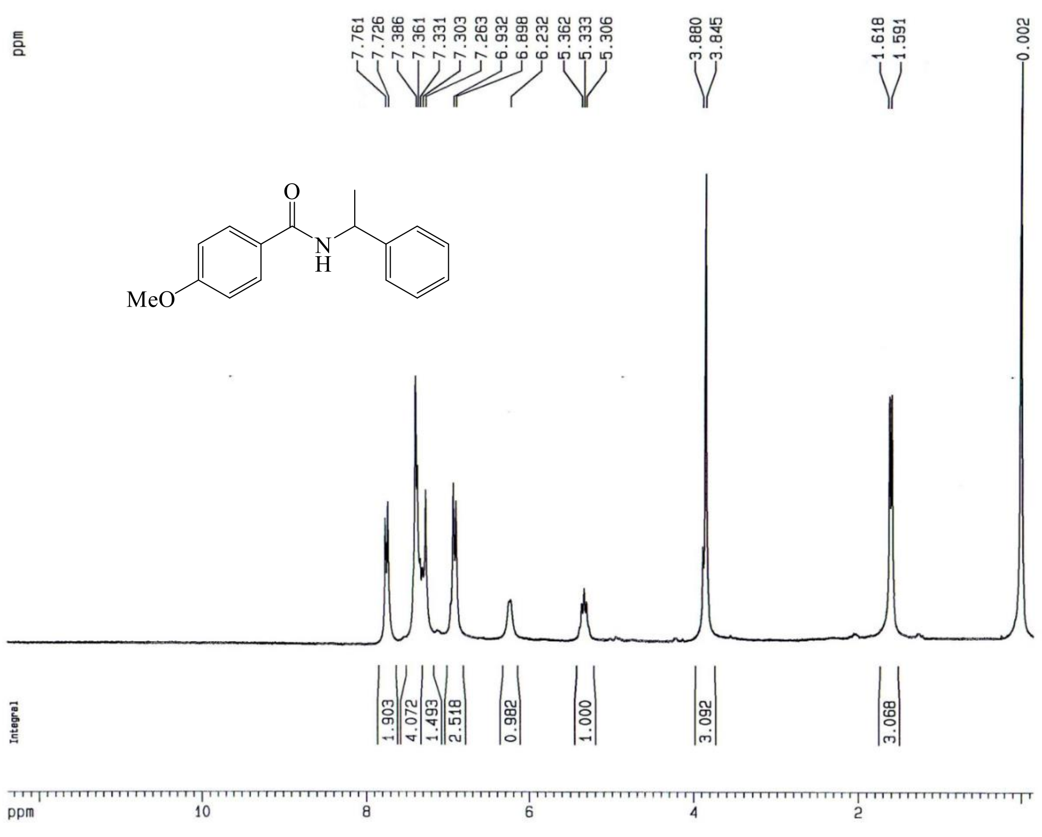
7.761
7.726
7.386
7.361
7.331
7.303
7.263
6.932
6.898
6.232
5.362
5.333
5.306
3.880
3.845
1.618
1.591
0.002
MeO
O
N
H
Integral
1.903
4.072
1.493
2.518
0.982
1.000
3.092
3.068
ppm
10
8
6
4
2

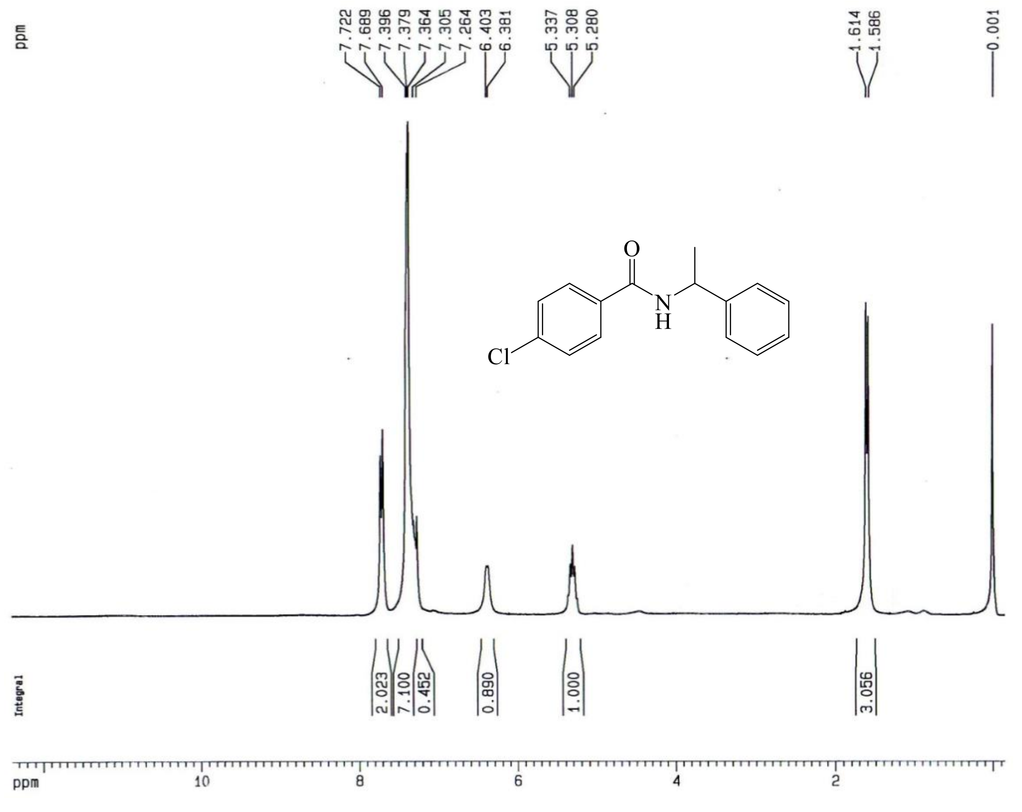
7.722
7.689
7.396
7.379
7.364
7.305
7.264
6.403
6.381
5.337
5.308
5.280
1.614
1.586
0.001
Cl
O
N
H
Integral
2.023
7.100
0.452
0.890
1.000
3.056
ppm
10
8
6
4
2

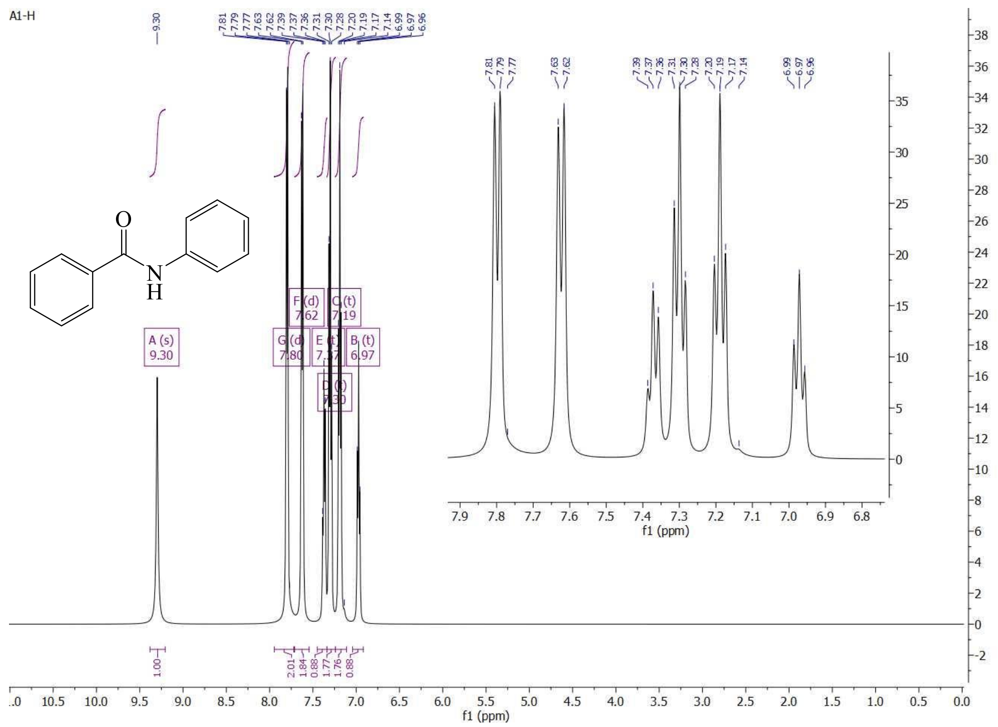
A1-H
A (s)
9.30
G (d)
7.80
F (d)
7.62
E (t)
7.37
D (t)
7.30
C (t)
7.19
B (t)
6.97
f1 (ppm)

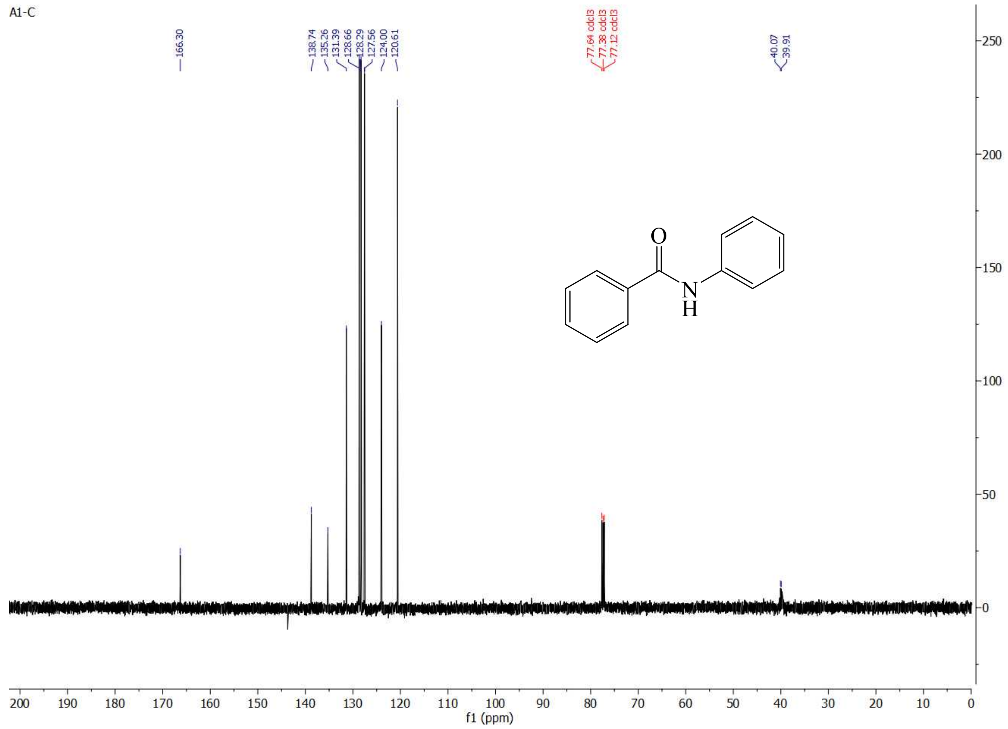
A1-C
166.30
138.74
135.26
131.39
128.66
128.29
127.56
124.00
120.61
77.64 cdcl3
77.38 cdcl3
77.12 cdcl3
40.07
39.91
f1 (ppm)

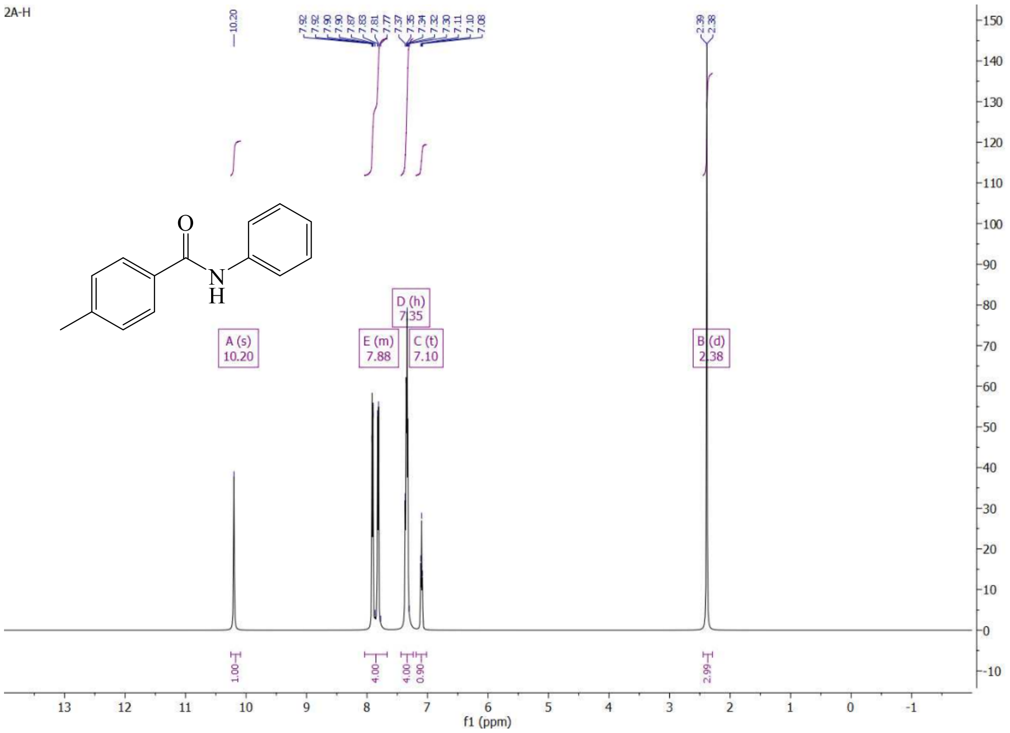
2A-H
A (s) 10.20
E (m) 7.88
D (h) 7.35
C (t) 7.10
B (d) 2.38
f1 (ppm)

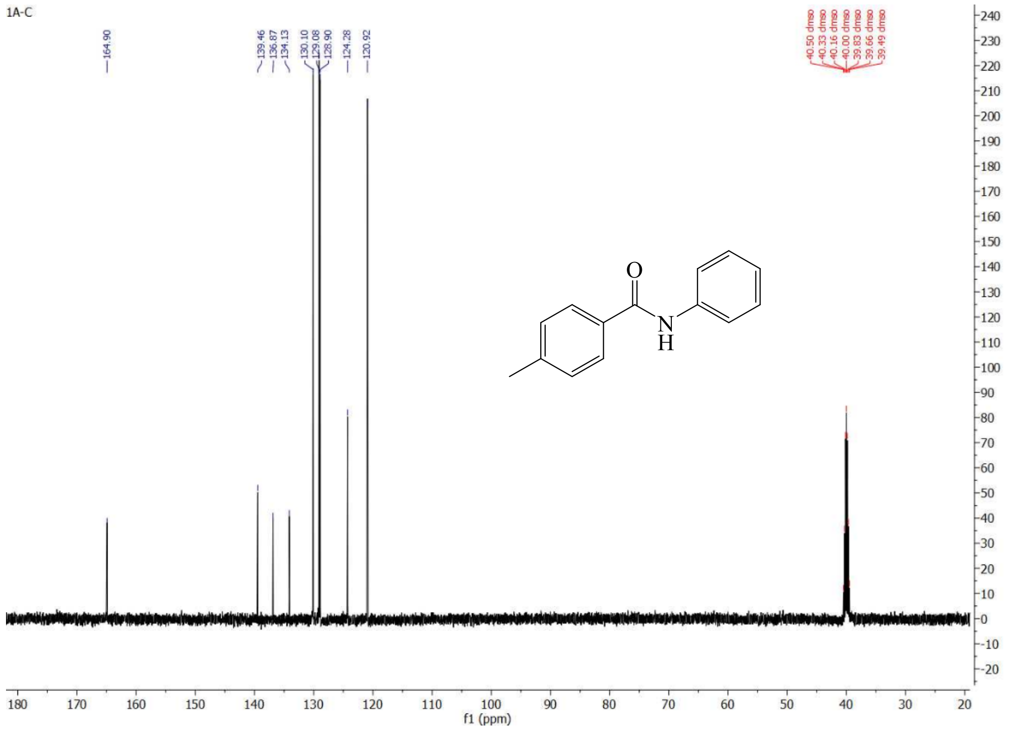
1A-C
164.90
139.46
136.87
134.13
130.10
129.08
128.90
124.28
120.92
f1 (ppm)

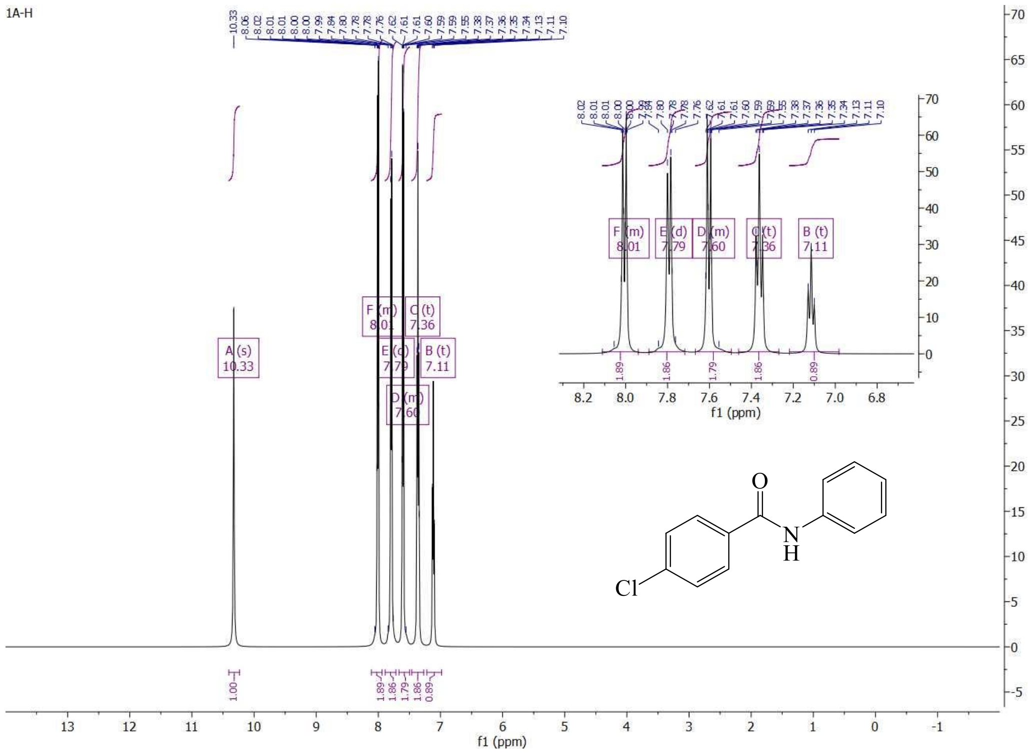

1A-H
10.33
8.06 8.02 8.01 8.01 8.00 8.00 7.99 7.84 7.80 7.78 7.78 7.76 7.62 7.61 7.61 7.60 7.59 7.59 7.55 7.38 7.37 7.36 7.35 7.34 7.13 7.11 7.10
A (s) 10.33
F (m) 8.01
E (d) 7.79
D (m) 7.60
C (t) 7.36
B (t) 7.11
1.00
1.89
1.86
1.79
1.86
0.89
O
N
H
Cl
f1 (ppm)

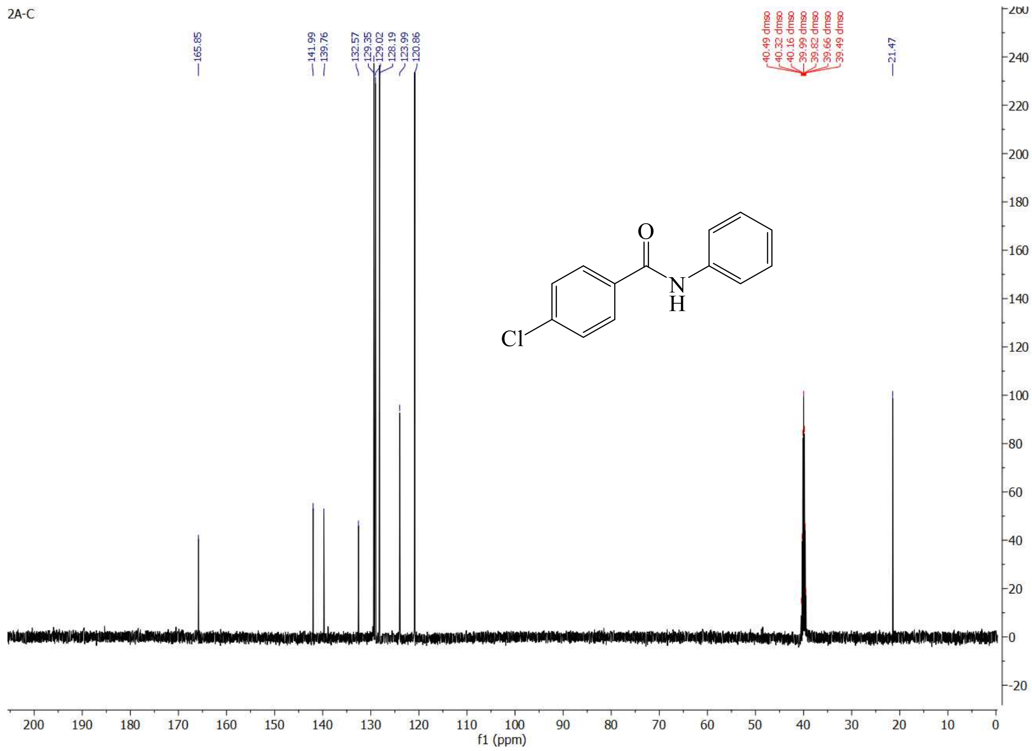

2A-C
165.85
141.99
139.76
132.57
129.35
129.02
128.19
123.99
120.86
40.49 dmso
40.32 dmso
40.16 dmso
39.99 dmso
39.82 dmso
39.66 dmso
39.49 dmso
21.47
O
N
H
Cl
f1 (ppm)

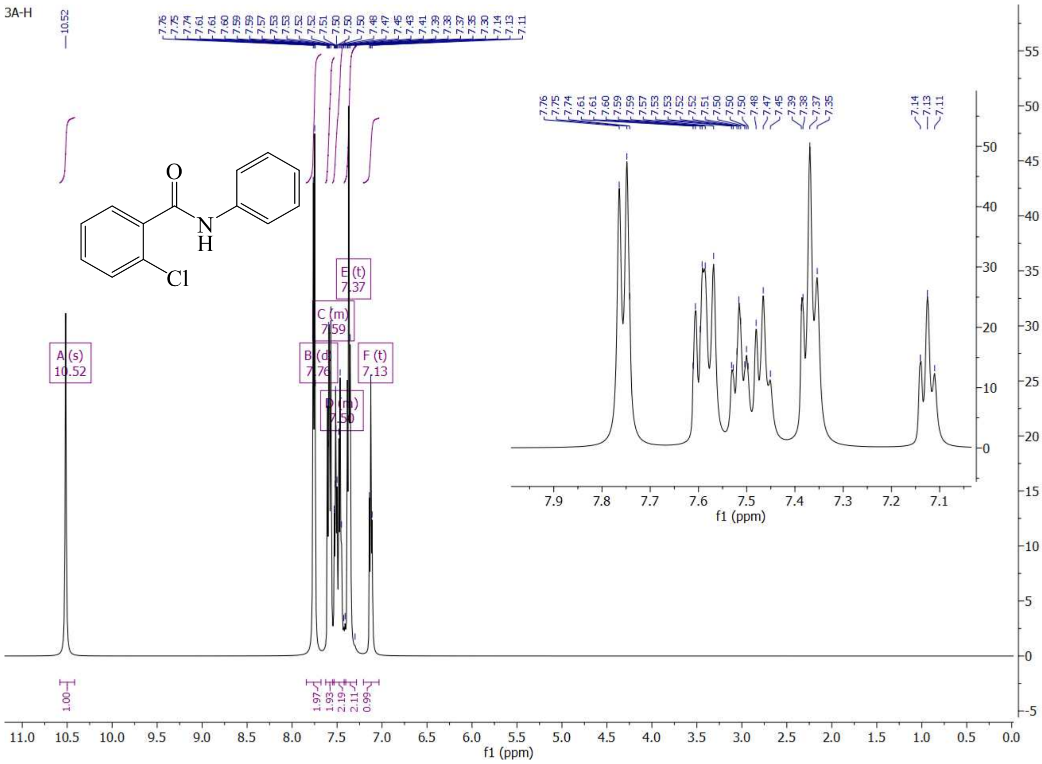
3A-H
A (s)
10.52
B (d)
7.76
C (m)
7.59
D (m)
7.50
E (t)
7.37
F (t)
7.13
f1 (ppm)

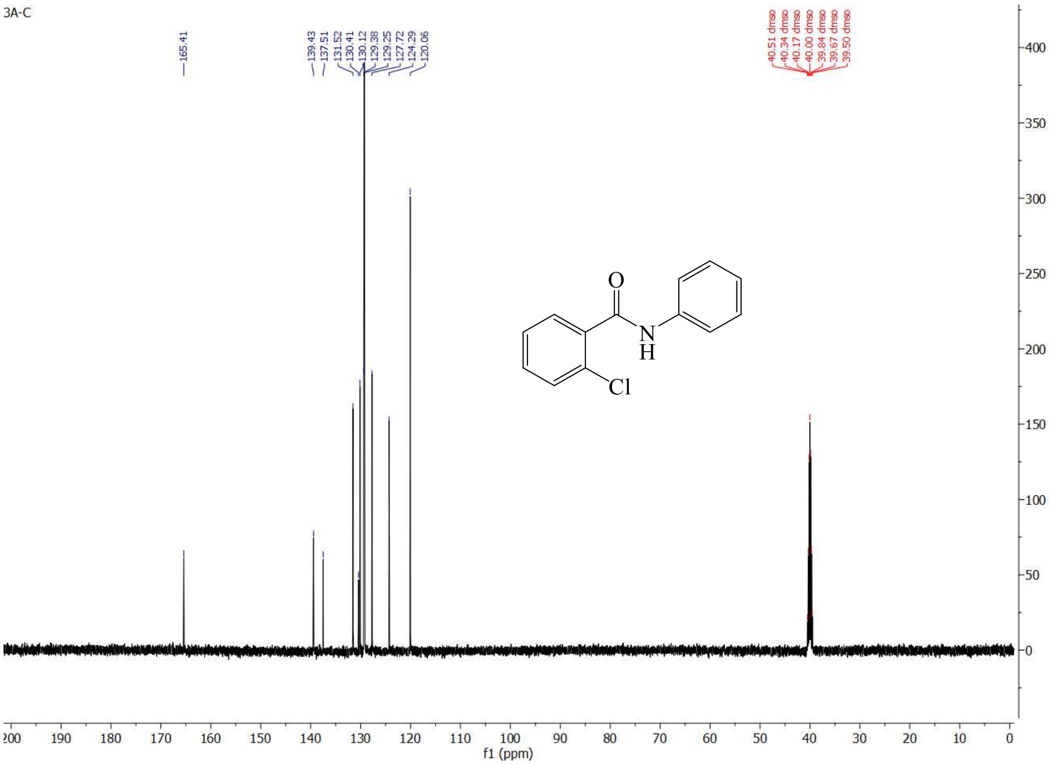
3A-C
f1 (ppm)

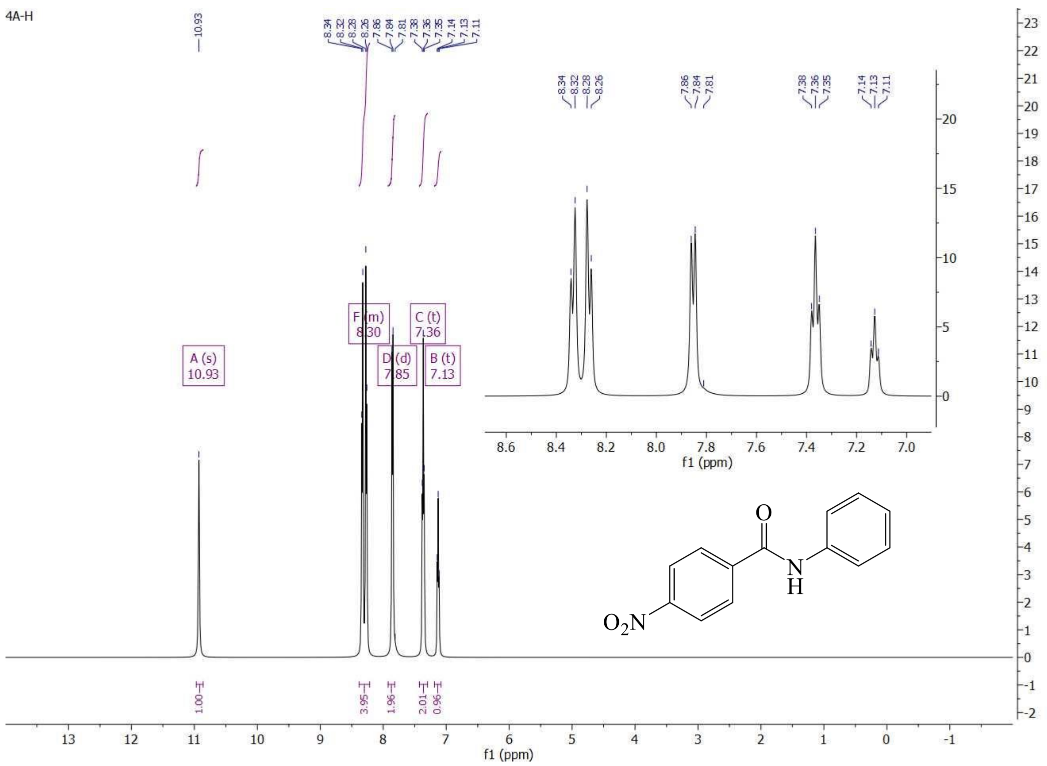
4A-H
A (s)
10.93
F (m)
8.30
D (d)
7.85
C (t)
7.36
B (t)
7.13
f1 (ppm)
O
N
H
O2N

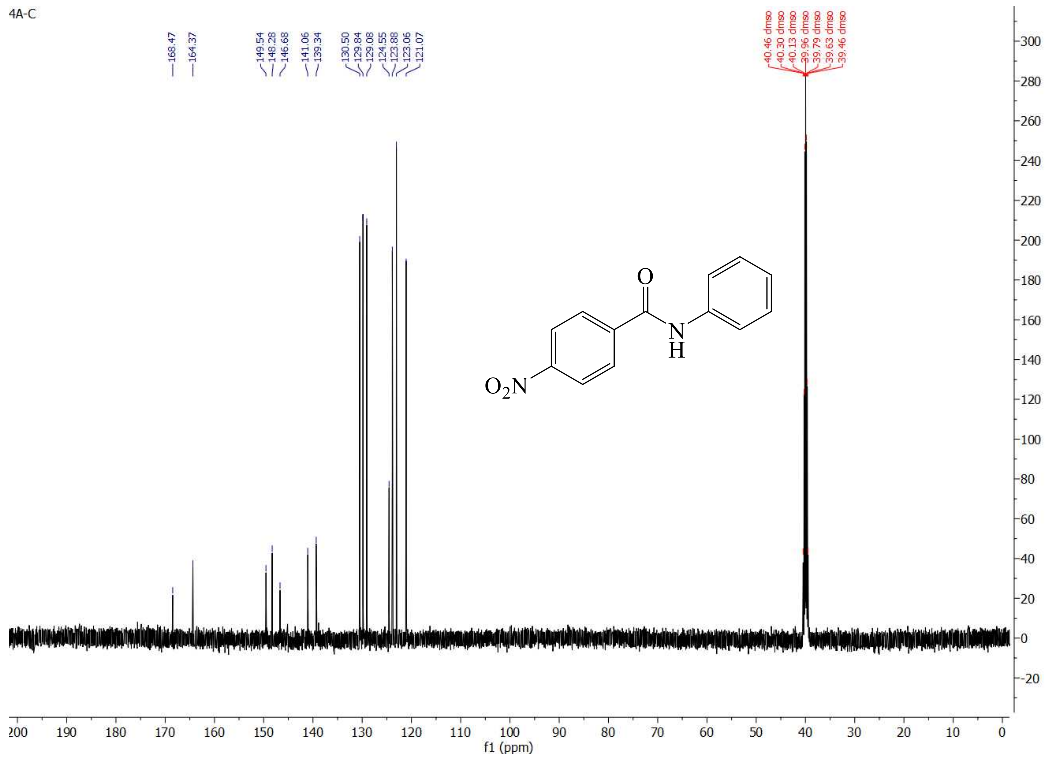
4A-C
f1 (ppm)
O
N
H
O2N

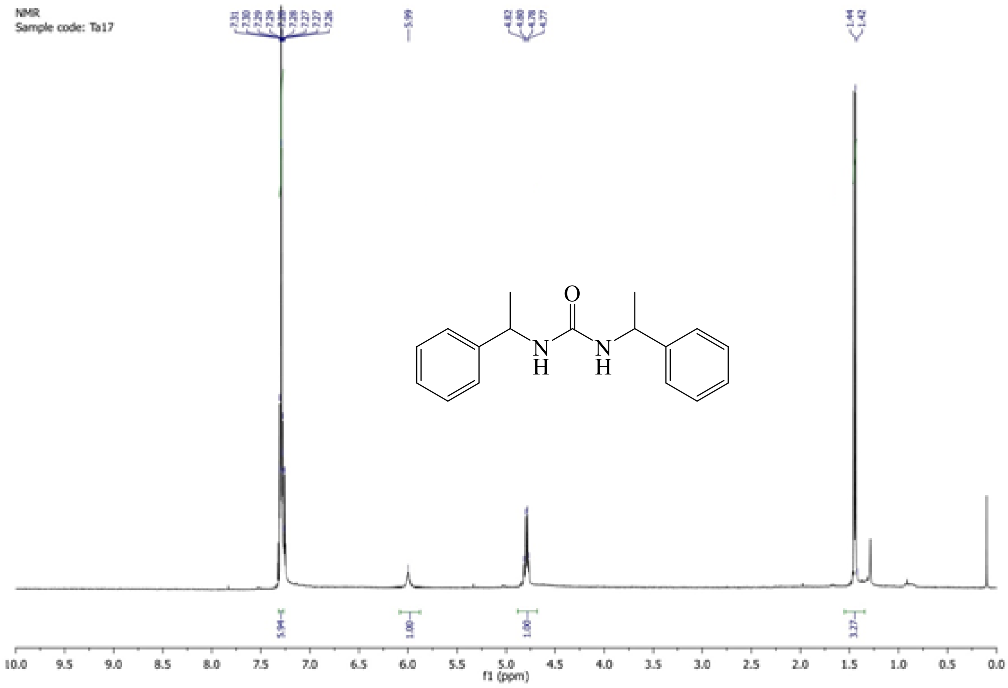
NMR
Sample code: Ta17
O
N
H
N
H
f1 (ppm)

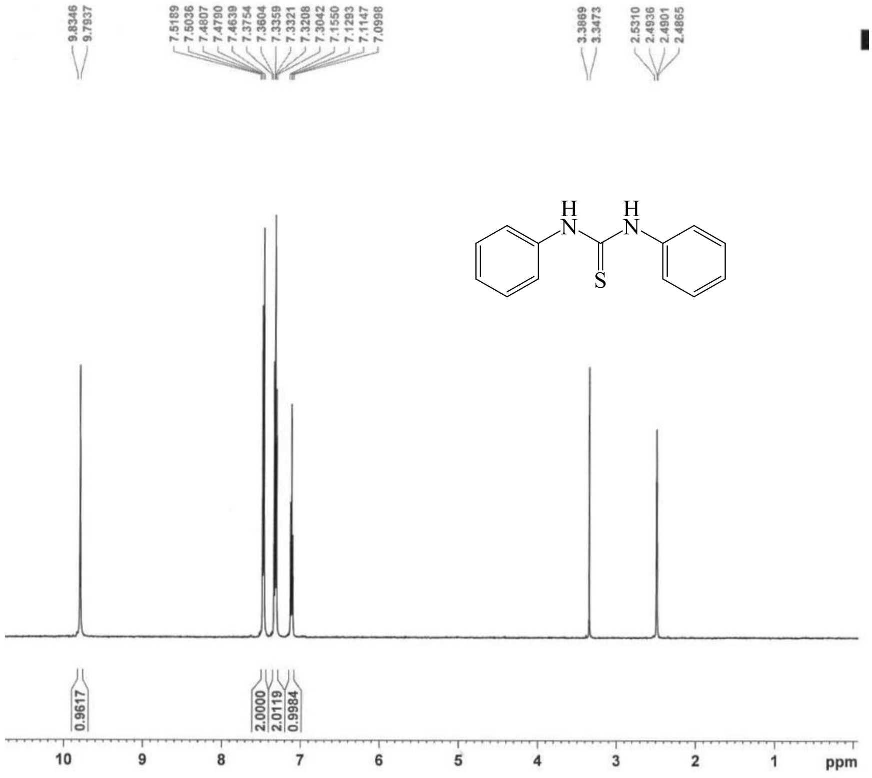
9.8346
9.7937
7.5189
7.5036
7.4807
7.4790
7.4639
7.3754
7.3604
7.3359
7.3321
7.3208
7.3042
7.1550
7.1293
7.1147
7.0998
3.3869
3.3473
2.5310
2.4936
2.4901
2.4865
H
N
H
N
S
0.9617
2.0000
2.0119
0.9984
ppm
